# Targeting the CXCR4/CXCL12 Axis to Overcome Drug Resistance in Triple-Negative Breast Cancer

**DOI:** 10.3390/cells14181482

**Published:** 2025-09-22

**Authors:** Desh Deepak Singh, Dharmendra Kumar Yadav, Dongyun Shin

**Affiliations:** 1 Amity Institute of Biotechnology, Amity University Rajasthan, Jaipur 303002, India; ddsbms@gmail.com; 2 College of Pharmacy, Gachon University, Hambakmoeiro 191, Yeonsu-gu, Incheon 21924, Republic of Korea

**Keywords:** triple-negative breast cancer (TNBC), CXCR4, CXCL12, drug resistance, chemoresistance, targeted therapy

## Abstract

Triple-negative breast cancer (TNBC) remains one of the most aggressive and treatment-resistant forms. TNBC is an aggressive and therapeutically resistant subtype of breast cancer, marked by the absence of estrogen, progesterone, and HER2 receptors. The lack of defined molecular targets significantly limits treatment options and contributes to high recurrence rates. Among the key pathways involved in TNBC progression and resistance, the CXCR4/CXCL12 chemokine axis has emerged as a critical player. CXCR4, a G-protein-coupled receptor, binds specifically to its ligand CXCL12, promoting tumour cell proliferation, metastasis, immune evasion, and stromal remodelling. Its overexpression is frequently associated with poor prognosis, disease progression, and resistance to conventional therapies in TNBC. This review explores how the chemokine receptor type 4 (CXCR4/CXCL12) axis facilitates drug resistance through mechanisms such as epithelial–mesenchymal transition (EMT), cancer stemness, and microenvironmental interactions. Notably, CXCR4 antagonists like plerixafor, balixafortide, and POL5551 have shown encouraging preclinical and clinical results, particularly when combined with chemotherapy or immunotherapy. Additionally, innovative strategies, including radiopharmaceuticals, peptide inhibitors, and nanotechnology-based delivery platforms, offer expanded therapeutic avenues. Despite persistent challenges such as tumour heterogeneity and potential toxicity, growing clinical evidence supports the translational relevance of this axis. This manuscript provides an in-depth analysis of CXCR4/CXCL12-mediated drug resistance in TNBC and evaluates current and emerging therapeutic interventions.

## 1. Introduction

**Figure 1:**
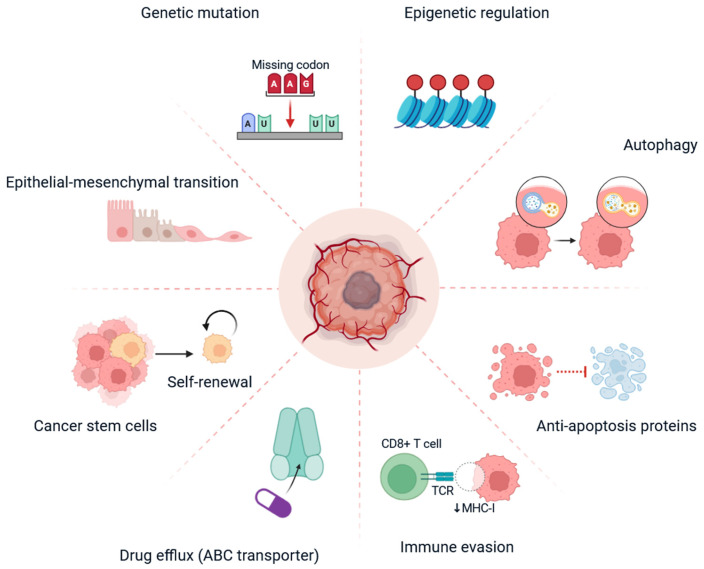
This diagram illustrates the multifactorial pathways leading to drug resistance in TNBC. At the centre of this process is the capacity of the tumour cell to survive and adapt to therapeutic stress (Biorender.com accessed on 17 July 2025).

Triple-negative breast cancer (TNBC) accounts for approximately 10–20% of all breast cancer cases and is defined by the absence of established hormone receptors, including estrogen receptor (ER), progesterone receptor (PR), and human epidermal growth factor receptor 2 (HER2) [1]. It is known for greater aggressiveness, the ability to metastasise early, and the tendency to recur early, and it has a historically poor prognosis [2]. With a lack of targetable ER, PR, or HER2, systemic chemotherapy is the only treatment modality available to patients [3]. Major mechanisms involved include genetic mutations (e.g., in tumour suppressor genes or oncogenes), epigenetic modulation (e.g., histone modifications), and autophagy, which enables cancer cells to recycle nutrients under stress conditions [4]. Epithelial–mesenchymal transition (EMT) and the expression of anti-apoptotic proteins facilitate invasion and resistance by preventing programmed cell death. Immune evasion, such as the downregulation of MHC-I, impairs immune system recognition [5]. Drug efflux via ATP-binding cassette (ABC) transporters reduces intracellular drug concentrations. Cancer stem cells contribute through self-renewal and resistance to conventional therapies [6]. Individually or in combination, these mechanisms enable TNBC cells to survive chemotherapy, posing significant challenges to effective treatment (Figure 1) [7]. TNBC patients frequently develop chemoresistance, contributing to treatment failure and disease recurrence [8]. There is an increasing body of evidence to indicate that the tumour microenvironment (TME), being made up of immune cells, stromal components and a milieu of signalling molecules, is a significant player in altering drug response and enabling resistance mechanisms [9]. The chemokine receptor CXCR4 and its ligand CXCL12 (also referred to as stromal-derived factor-1, SDF-1) have become prominent in TNBC progression and drug resistance [10]. CXCR4 is a G-protein-coupled receptor (GPCR) that is overexpressed in many solid tumours, including TNBC [11]. Its sole ligand, CXCL12, is secreted by stromal cells (cancer-associated fibroblasts (CAFs), mesenchymal stem cells, and endothelial cells) in the tumour microenvironment (TME) [12]. Upon binding of CXCL12 with CXCR4, signalling events activate multiple downstream signalling pathways, including phosphoinositide 3-kinase (PI3K)/AKT, mitogen-activated protein kinase (MAPK/ERK), and janus kinase (JAK)/signal transducers and activators of transcription (STAT) pathways, facilitating tumour cell survival, proliferation, migration, and immune evasion [13]. The CXCR4/CXCL12 axis certainly facilitates tumour cell homing, specifically in organs such as the lung, liver, bone, and lymph nodes, which are the most common sites of TNBC metastasis [14]. An essential feature of CXCR4/CXCL12 signalling in TNBC is its ability to mediate resistance to chemotherapy and targeted treatment [15]. Mechanistically, activation of CXCR4 leads to the transition to the epithelial–mesenchymal transition (EMT), enhancement of cancer stem cell (CSC) properties, and establishment of a pro-survival phenotype [16]. EMT is characterised by the downregulation of epithelial markers and upregulation of mesenchymal characteristics that allow tumour cells to be more migratory, invasive, and resistant to apoptosis [17]. Additionally, CXCR4/CXCL12 signalling enhances CSC populations to mediate resistance by retaining a portion of quiescent, drug-tolerant cells, which are capable of repopulating the tumour after treatment is paused [18]. In preclinical models, CXCR4 inhibition has been shown to re-sensitize TNBC cells to chemotherapeutic agents, including doxorubicin, paclitaxel, and cisplatin [15]. Even without combining with chemotherapy, pharmacologic blockade using CXCR4 antagonists, such as AMD3100 (plerixafor), AMD3465, or through monoclonal antibodies, has demonstrated the ability to reduce tumour burden, delay metastasis, and enhance chemosensitivity in preclinical TNBC models [19]. Additionally, CXCR4 blockade combined with standard chemotherapy produced a synergistic anti-tumour effect, underscoring the axis’s potential as a therapeutic target [20]. More than intrinsic tumour cell behaviour, CXCR4/CXCL12 signalling contributes to the immune suppressive microenvironment seen in TNBC by recruiting regulatory T cells (Tregs), myeloid-derived suppressor cells (MDSCs), and tumour-associated macrophages (TAMs) to the tumour, creating a block to tumour-specific immunity and promoting tumour evasion of the immune system. Moreover, CXCR4/CXCL12 signalling inhibits CD8^+^ T cell infiltration while decreasing CD8^+^ T cell functionality, which inhibits the efficacy of immune checkpoint inhibitors (ICIs), including therapeutic products targeting PD-1 and PD-L1 [21]. Thus, there is an expanding interest in CXCR4 blockade combined with immunotherapy. Recently initiated clinical trials have started exploring this combination in an attempt to “recondition” the tumour microenvironment (TME) to eliminate immune resistance to immunotherapeutic products in patients with TNBC [22]. In addition, CXCL12 expression is further upregulated by hypoxia in the TME through hypoxia-inducible factor-1α (HIF-1α), leading to further CXCR4-related signalling and chemoresistance [23]. This ultimately forms a self-amplifying loop where chemotherapy-induced hypoxia leads to unintentional CXCR4/CXCL12 activation and consequently tumour regrowth [13]. Clearly, this presents significant implications for the timing and combinations of therapy where CXCR4 inhibitors could be advantageous for cancer care by interrupting this self-amplifying loop before it could take root [24]. Novel drug delivery approaches (e.g., nanoparticles and liposomes) are also being utilised to co-deliver agents targeting CXCR4 and chemotherapy directly to the tumour site, which ameliorates systemic toxicities and increases efficacy [25]. Precision oncology can identify TNBC patients with high CXCR4/CXCL12 expression, enabling targeted therapies to improve treatment outcomes [26]. Biomarker-step clinical trials are now in progress for the purpose of stratification of patients and treatment regimens. Despite these positive developments, important obstacles must be addressed [27]. CXCR4 exerted some significant physiologic effects during haematopoiesis, vascular cardiac development, immune cell trafficking, etc. [10]. Concerns exist about systemic toxicities and off-target effects attributed to CXCR4 inhibition. In general, due to compensatory signalling via other chemokine receptors (e.g., CXCR7), the benefit derived from CXCR4 inhibition could be mitigated in the other adverse event of malignant disease for therapeutic development [28].

TNBC does not have effective targeted therapies and is more likely to develop chemoresistance, which leads to high rates of poor clinical outcomes [10,11,12]. One of the main players in this resistance is the CXCR4/CXCL12 signalling axis that is known to play a role in promoting tumour progression, metastasis, survival and remodelling the tumour microenvironment [8,9,10,11,12]. The dysregulation of this pathway leads to activation of multiple downstream signalling cascades, such as PI3K/AKT, MAPK, and JAK/STAT, which provide tumour cells with increased cellular plasticity when confronted with therapeutic stress [13]. The goal of this review was to connect mechanistic understanding with translational/clinical aspects, while highlighting targeting of the CXCR4/CXCL12 axis as a novel therapeutic approach to overcome drug resistance in TNBC (Figure 1) [25,26,27,28]. Therefore, in order for rational drug design to develop durable benefits with therapeutic benefits along with safety and adverse event tolerability, true combination approaches are necessary. The CXCR4/CXCL12 axis is crucial in TNBC aggressiveness, metastasis, and therapy resistance [29]. Therapeutic strategies to interfere with this axis have produced promising preclinical and early clinical results [30]. Current investigations should evolve these approaches, taking into account molecular information, efficient drug delivery, and combinations to overcome resistance patterns of drugs and improve clinical responses of patients with TNBC [30]. Comprehensive knowledge of this axis, in the breadth of the tumour ecosystem, could reshape TNBC management and potentially allow for a paradigm shift in the management of drug resistance in this challenging subtype of cancer. This manuscript discusses the clinical challenges in TNBC and drug resistance, the mechanism of the CXCR4/CXCL12 axis in drug resistance, strategies to overcome CXCR4/CXCL12-mediated drug resistance in TNBC, clinical trials aimed at overcoming drug resistance in TNBC, limitations of CXCR4/CXCL12-targeted therapy, and future directions for targeting the CXCR4/CXCL12 axis in TNBC (Figure 1).

## 2. Triple-Negative Breast Cancer and Drug Resistance: A Clinical Challenge

**Figure 2:**
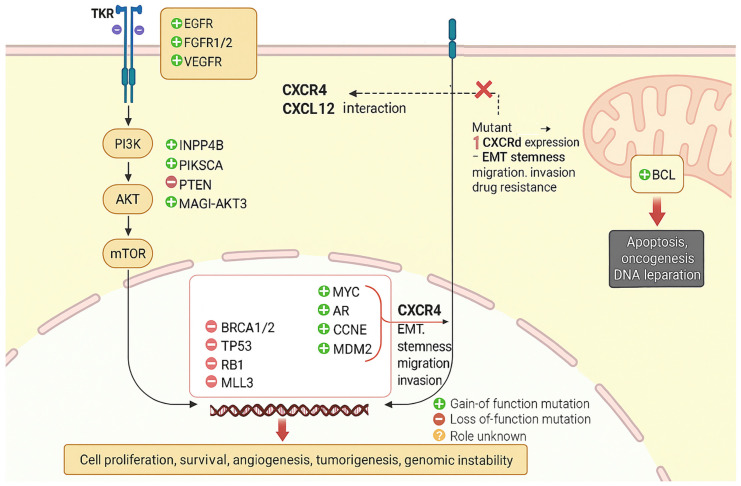
This diagram provides an overview of key signalling and genetic changes involved in TNBC. Binding of growth factors (EGFR, FGFR1/2, VEGFR) to receptor tyrosine kinases (TKRs) activates the PI3K–AKT–mTOR pathway, driving cell proliferation, survival, angiogenesis, tumourigenesis, and genomic instability. Gain-of-function mutations (e.g., INPP4B, PIKSCA, MAGI-AKT3, MYC, AR, CCNE, MDM2) and loss-of-function mutations (e.g., PTEN, BRCA1/2, TP53, RB1, MLL3) further dysregulate these processes. CXCR4–CXCL12 interaction promotes epithelial–mesenchymal transition (EMT), stemness, migration, invasion, and drug resistance. Mutations leading to upregulated CXCR4 expression amplify these oncogenic features. BCL activation inhibits apoptosis and alters DNA repair. These signalling alterations contribute to cancer progression and therapeutic resistance (Biorender.com).

The diversity of TNBC, the lack of hormonal receptors, and less effective targeted therapy greatly increase the chance for early relapse, increase metastatic potential, and diminish overall survival in comparison to other breast cancer subtypes [3]. TNBC has distinct clinical and pathological characteristics [3]. The tumours are predominantly high grade, have a basal-like genomic expression profile and have a high proliferative index [31]. In contrast to hormone receptor-positive or HER2-amplified cancers, there are no effective molecular targets for the treatment of TNBC [2]. A number of genetic markers are involved in this resistance [32]. BRCA1/2 mutations, although originally sensitising the tumours to DNA-damaging drugs and PARP inhibitors, are able to develop reversion mutations that recreate DNA repair and drug resistance [33]. TP53 mutations, which occur in more than 80% of TNBCs, induce genomic instability and apoptosis-evading resistance [34]. In addition to disrupting canonical tumour suppressor pathways, we hypothesise that TP53 mutations may impact chemokine signalling networks, altering CXCR4 expression and activity. In summarising this p53–CXCR4/CXCL12 relationship, we describe the potential novel mechanism of therapeutic resistance [34]. In addition, the review brings together newer preclinical evidence that targeting CXCR4 or its downstream pathways can sensitise TNBC cells to chemotherapy and immunotherapies [34,35]. Clinically, the evidence from ongoing studies targeting CXCR4 antagonism underscores the clinical relevance of this axis. Integrating mechanisms and therapeutic implications, this review encapsulates a large breadth of information to provide an updated and broad view of how targeting the p53–CXCR4/CXCL12 axis may help to overcome resistance and improve both outcomes for patients with TNBC [34,35]. PIK3CA mutations and PTEN loss activate the PI3K/AKT/mTOR pathway and lead to survival signalling and therapy evasion [35]. Androgen receptor (AR) overexpression is linked to chemo resistance and defines a unique TNBC subgroup [14]. Overexpression of CXCR4, usually triggered by genetic and epigenetic changes, enhances metastasis and chemoresistance by activating survival signals and drug efflux pump pathways (Table 1) [36]. Familiarity with these genetic markers is central to the anticipation of therapeutic response, the creation of focused interventions, and the repulsion of drug resistance in TNBC (Figure 2) [37]. Thus, in comparison to other metastatic breast cancers, systemic chemotherapy is the principal treatment modality for TNBC [38]. Along with the standard first line of treatment using various combinations of anthracyclines, taxanes, or platinum-based regimens generally on a quasi-weekly basis, many patients with metastatic TNBC will have an initial response to chemotherapy, but most patients will develop the progressive disease at a later time due to the development of drug resistance. Patients who have TNBC are also more likely than other intrinsic cancer types to develop visceral and brain metastases, which makes the patient more difficult to treat. The objective of this review is to offer a translational overview that integrates mechanistic insights with clinical perspectives, thereby highlighting the therapeutic relevance of targeting the CXCR4/CXCL12 axis to overcome drug resistance in TNBC management. Triple-negative breast cancer poses a significant clinical challenge due to its high rate of drug resistance, as shown in Table 1 [39,40,41,42,43,44,45].

## 3. Mechanisms of Drug Resistance in TNBC

**Figure 3:**
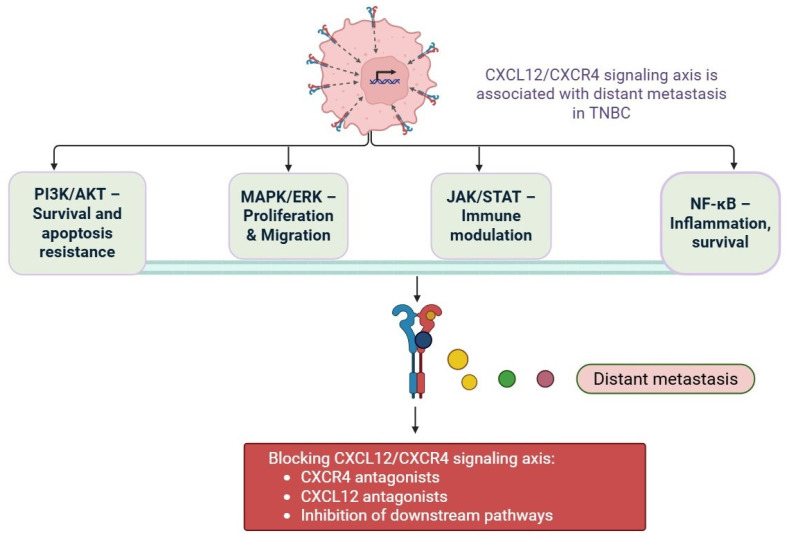
CXCL12 secreted from stromal and endothelial cells binds to CXCR4 on TNBC cells, activating downstream pathways (PI3K/AKT, MAPK/ERK, STAT3, NF-κB) that promote epithelial–mesenchymal transition (EMT), survival, invasion, and ultimately distant metastasis (Biorender.com).

Drug resistance in TNBC is rooted in both intrinsic and acquired mechanisms. A range of molecular and cellular processes contribute to chemotherapy failure [46]. One of those processes is EMT, which is a biological process characterised by epithelial tumour cells assuming mesenchymal properties, providing those cells greater motility, invasiveness, and likelihood of evading apoptosis [47]. TNBC cells that undergo EMT are more likely to survive chemotherapeutic agents’ cytotoxic effects [48]. TNBC is also enriched for cancer stem cell-like populations that are self-renewing, tumour-initiating, and resistant to conventional therapies [49]. Cancer stem cells can often survive chemotherapy and then repopulate the tumour, leading to a relapse. Other factors contributing to TNBC drug resistance include overexpression of ATP-binding cassette (ABC) transporters such as P-glycoprotein (P-gp/ABCB1), which can actively pump chemotherapeutic agents away from the cancerous or tumour cell, limiting drug accumulation in the cell [50]. TNBC boasts significant defects in homologous recombination (HR). This is most evident in the BRCA-mutated cases [51]. Though such tumours will respond favourably to agents that damage DNA, such as platinum compounds or PARP inhibitors, they may develop secondary mutations that restore HR proficiency [52]. The drug resistance landscape in TNBC is quite complicated. The tumour microenvironment (TME), which includes immune cells, fibroblasts, cytokines, and components of the extracellular matrix, is an important part of this landscape [9]. The tumour microenvironment has signals also derived from the tumour microenvironment, such as CXCL12, TGF-β, and IL-6, that can promote tumour survival and chemoresistance [53]. The drug-resistant phenotype in TNBC is also influenced by the dysregulation of epigenetic modulators, the activation of signalling pathways (e.g., PI3K/AKT, NF-κB, and Wnt/β-catenin), and other cellular mechanisms [54]. There are currently ongoing studies that are looking at whether agents targeting epigenetic modulators or agents that can eliminate cells that have undergone an EMT or cancer stem cell (CSC) characteristics (e.g., Notch inhibitors, Hedgehog inhibitors or Wnt inhibitors) could form the basis of combination therapy [55]. Immune checkpoint inhibitors, such as PD-1/PD-L1 inhibitors, will also have effects upon TME in the normal immune system and may also have importance as agents for TNBC, along with compatible and previously unassigned treatments like immunomodulatory agents such as chemotherapy [55]. PD-1/PD-L1 inhibitors have already had preliminary testing in TNBC with other therapeutic combinations, while mixed signals about success have come from the use of these treatments now approved for use in TNBC (i.e., PARP inhibitors olaparib and talazoparib for BRCA genetic-mediated TNBC). More broadly, these PARP inhibitors could be explored for use on the wider populations and considered for use once again, along with other popular agents of therapies provided in mixed treatments such as targeted inhibitors [52]. Most importantly, preclinical studies suggest that the pharmacological inhibition of PARP not only affects DNA repair but also inhibits CXCL12/CXCR4 signalling, which influences tumour cell migration, reduces metastatic ability, and potentially increases therapeutic response [52]. Combination therapy approaches with PARP inhibitors plus CXCR4 antagonists, neutralizing antibodies, or downstream signalling inhibitors may offer synergistic clinical benefit by disrupting DNA repair capability and simultaneously blocking pro-survival signal pathways [53,54]. This may provide a multi-faceted strategy to defeat drug resistance and/or prevent distant metastasis, and may ultimately lead to improved therapeutic outcomes for patients with TNBC, which remain a subtype without any effective targeted therapies [54]. The CXCR4/CXCL12 axis is a crucial mechanism in cancer progression, especially in TNBC [56]. Stromal cells in the tumour microenvironment secrete CXCL12, which in turn binds to the CXCR4 receptor on the cancer cell and activates many signalling pathways (e.g., PI3K/AKT and MAPK) that promote tumour cell proliferation, tumour cell migration, the EMT, and even metastasis (Figure 3) [57]. Other features of the CXCR4/CXCL12 signalling pathway include being a contributor to drug resistance by enriching the cancer stem cells (CSCs) and creating an immunosuppressive surrounding microenvironment. CXCR4/CXCL12 also facilitates the homing of tumour cells to distal organs while limiting therapeutic effectiveness due to protecting tumour cells from immune attack and apoptosis, making it an important therapeutic target [58].

## 4. Mechanism of CXCR4/CXCL12 Axis in Drug Resistance in Triple-Negative Breast Cancer

**Figure 4:**
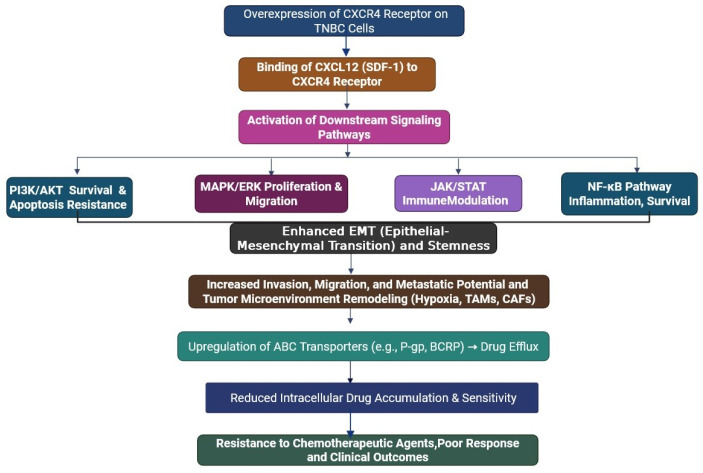
This flowchart shows how the molecular mechanism involved in CXCR4/CXCL12 (SDF-1) signalling axis leads to drug resistance of TNBC. Hyperexpression of CXCR4 receptors by TNBC cells, upon stimulation by CXCL12, triggers multiple downstream signalling pathways, such as PI3K/AKT (survival and apoptosis resistance), MAPK/ERK (proliferation and migration enhancement), JAK/STAT (immune modularity), and NF-κB (inflammation and survival). These mechanisms together promote epithelial–mesenchymal transition (EMT) and stemness, leading to enhanced invasion, migration, and metastatic activity, as well as tumour microenvironment remodelling (e.g., hypoxia, TAMs, CAFs). This in turn results in the up-regulation of ATP-binding cassette (ABC) drug transporters, including P-gp and BCRP, which can efflux chemotherapeutics and minimise intracellular drug concentration. As a result, TNBC cells display resistance to chemotherapeutic drugs, a lack of treatment response, and poor clinical outcomes.

TNBCs often develop resistance to chemotherapy agents, causing recurrence and poor survival. One of the important molecular pathways mediating chemotherapy resistance is the CXCR4/CXCL12 chemokine axis [59]. CXCR4 is a G-protein-coupled receptor (GPCR) expressed in many different tumour cells, including TNBC. CXCR4 has a unique ligand known as CXCL12 (also known as stromal-derived factor-1 or SDF-1), which is secreted from multiple stromal components in the TME, such as cancer-associated fibroblasts (CAFs), mesenchymal stem cells and endothelial cells (Figure 4) [57]. Upon binding CXCR4, CXCL12 promotes multiple downstream signalling cascades that can facilitate tumour survival/progression and even resistance (Table 2) [60]. Additionally, the CXCR4/CXCL12 axis has an important role in the remodelling of the tumour microenvironment (TME) [57]. It promotes the recruitment of immune and stromal cells, promotes angiogenesis, enhances interaction between cancer cells and the surrounding niche, and modifies the environment to protect cancer cells by shielding them from therapeutic insults. Frequency of immune suppression, particularly in the form of increased regulatory T-cell (Treg) recruitment, again limits the effectiveness of immunotherapies for TNBC [60]. Together, the CXCR4/CXCL12 axis promotes drug resistance in the tumour primarily in a twofold manner: (1) direct tumour cell survival mechanisms and enrichment of resistant CSC populations, and (2) modifying the TME. One has to consider the CXCR4/CXCL12 axis as another way to leverage therapeutic efficacy in TNBC.

The key mechanisms through which the CXCR4/CXCL12 axis promotes drug resistance are discussed below.

## 5. Activation of Survival

Upon binding of CXCL12 to CXCR4, CXCR4 activates key intracellular signalling pathways, including PI3K/AKT, MAPK/ERK, JAK/STAT, and NF-κB, that promote proliferation and survival/suppress apoptosis signalling processes [13,64,65]. By positively influencing the expression of anti-apoptotic proteins (e.g., Bcl-2, survivin), while negatively influencing pro-apoptotic cellular processes, CXCR4 signalling allows TNBC to dodge the cytotoxic effects of chemotherapeutics, such as doxorubicin and paclitaxel [66]. The CXCL12/CXCR4 axis drives survival and leads to drug resistance due to the activation of multiple pro-survival signalling cascades in TNBC [13]. Upon binding of the chemokine ligand CXCL12, the receptor CXCR4 is activated, which in turn activates downstream signalling pathways, including the PI3K/AKT, MAPK/ERK, JAK/STAT, and NF-κB pathways [67]. These pathways then regulate important cellular functions, including proliferation, differentiation, and resistance to apoptosis. The activation of the PI3K/AKT and MAPK/ERK signalling pathways will lead to the phosphorylation of critical molecules that will enhance cell cycle progression and apoptosis inhibition [68]. Concurrently, the JAK/STAT signalling pathway will promote transcription of immune regulatory and survival genes, and NF-κB signalling will upregulate gene expression related to inflammatory and anti-apoptotic processes [69]. Together, the activation of CXCR4 signalling contributes to the regulation of apoptosis by promoting the expression of anti-apoptotic factors, such as Bcl-2 and survivin, while inhibiting pro-apoptotic factors, shifting the balance of apoptosis in favour of TNBC cells so that they can survive the cytotoxic impact of regimens used in therapeutic settings such as doxorubicin, paclitaxel, or cisplatin [59]. Activation of these signal pathways leads to the development of chemoresistance and tumour progression, thus highlighting the relevance of targeting CXCR4-mediated signalling in resistant TNBC with poor therapeutic response [11].

### 5.1. Induction of Epithelial–Mesenchymal Transition (EMT)

The CXCL12/CXCR4 axis is pivotal for triggering EMT, which is essential in drug resistance and tumour development in TNBC [57]. During EMT, epithelial cancer cells change their phenotype towards a mesenchymal-like morphology and develop increased motility, invasiveness, stemness, and resistance to apoptosis [70]. This change makes metastasis possible and leads to unfavourable clinical outcomes in patients with TNBC [71]. Following activation of the CXCR4 receptor by its ligand CXCL12, downstream signalling cascades are initiated, leading to the repression of epithelial markers, such as E-cadherin, and the induction of mesenchymal markers, including N-cadherin, vimentin, and transcription factors such as Snail, Slug, and Twist [72]. These molecular changes disrupt cell–cell adhesion, enhance cellular plasticity, and promote invasiveness. Additionally, EMT has been linked with chemoresistance, since mesenchymal-like cells have the ability to show increased DNA repair, decreased drug uptake, and apoptosis resistance to chemotherapeutic agents such as doxorubicin and paclitaxel [73]. TNBC cells treated with CXCL12 have consistently high EMT marker expression and demonstrate a strong, therapy-resistant phenotype [74]. Thus, targeting the CXCR4/CXCL12 axis is therapeutically promising in reversing EMT-related changes, improving chemosensitivity, and overcoming drug resistance in TNBC.

### 5.2. Maintenance and Expansion of Cancer Stem Cells (CSCs)

The CXCL12/CXCR4 signalling axis plays a critical, yet poorly understood, role in the maintenance and expansion of cancer stem cells (CSCs) in TNBC [75]. Cancer stem cells (CSCs) represent a small subpopulation of tumour cells that display self-renewal, differentiation potential, and significantly high resistance to conventional cytotoxic therapies [16]. CSCs have been shown to initiate tumours, metastasise tumour cells, promote recurrence of tumours, and contribute to failure of therapy [76]. Binding of CXCL12 to CXCR4 activates multiple downstream signalling pathways, like PI3K/AKT, JAK/STAT, and Wnt/β-catenin, which promote the survival and proliferation of CSCs [77]. This signalling pathway enhances expression of stemness markers, such as CD44^+^/CD24^−^, ALDH1, OCT4, SOX2, and NANOG, driving the stem-like phenotype in TNBC cells [78]. Most significantly, CSCs show profound resistance to chemo/radiation treatment because of their quiescence, increased DNA damage repair ability, efflux-mediated drug resistance, and inherent expression of anti-apoptotic proteins [79]. In addition, the activation of CXCR4 mediates protective effects for CSCs by maintaining a supportive niche that will provide physical shelter and immunity to therapeutic agents in the tumour microenvironment [80]. The enrichment of breast cancer CSCs through CXCL12/CXCR4 signalling is a contributor to prolonging tumour relapse and the propensity to metastasise post-treatment [81]. Therefore, targeting this axis not only inhibits the survival of CSCs, but in doing so may sensitise TNBC tumours to chemotherapy, discouraging relapse and thus enhancing durable control of their cancer.

### 5.3. Modulation of the Tumour Microenvironment and Immune Evasion

The CXCL12/CXCR4 axis is key to altering the TME and immune evasion in TNBC [13]. The TME contains many diverse stromal and immune cells, extracellular matrix components, and signalling molecules that together mediate tumour growth and quality of response to treatment [82]. In this environment, CXCL12, secreted by cancer-associated fibroblasts (CAFs) and mesenchymal stem cells, binds to the CXCR4 protein expressed on TNBC cells and immune cells, leading to the activation of signalling pathways, which promote an immunosuppressive tumour environment [83]. The activation of CXCR4 may recruit at least one of a number of immunosuppressive cells, including regulatory T cells (Tregs), myeloid-derived suppressor cells (MDSCs), and tumour-associated macrophages (TAM), that inhibit cytotoxic T cell responses and facilitate tumour growth [84]. Additionally, the CXCL12/CXCR4 axis restricts CD8^+^ cytotoxic T lymphocyte (CTL) infiltration and function, resulting in poor-quality responses to immune checkpoint inhibitors (ICIs), i.e., anti-PD-1 and anti-PD-L1 therapies [85]. In addition, CXCL12/CXCR4 signalling encourages angiogenesis and tissue remodelling, resulting in increased tumour vascularisation and increased potential for metastatic spread [86]. The chemokine-mediated immune suppression generates a phenotype that expresses the cold tumour phenotype that does not respond to immunotherapy [87]. Thus, disrupting the CXCL12/CXCR4 interactions provides a promising option for reprogramming the TME, enhancing immune surveillance and increasing the efficacy of immunotherapy for TNBC.

### 5.4. Promotion of Hypoxia-Induced Resistance

Hypoxia is a common feature of the tumour microenvironment in TNBC and contributes to therapy resistance, tumour aggressiveness, and metastasis [88]. In a hypoxic environment, the CXCL12/CXCR4 signalling axis is markedly upregulated, primarily through the action of hypoxia-inducible factor-1α (HIF-1α) [89]. HIF-1α enhances the transcription of CXCL12 in stromal cells and upregulates CXCR4 expression in TNBC cells, and therefore, the chemokine-receptor axis is heightened [10]. The subsequently enhanced CXCL12/CXCR4 signalling, under hypoxic conditions, activates downstream pathways, including PI3K/AKT and MAPK/ERK signalling, promoting cell survival, anti-apoptotic responses, and therapy resistance [81]. Additionally, hypoxia-mediated CXCR4 activation also mediates EMT and CSC persistence, both of which contribute to the drug-tolerant tumour phenotype [90]. The hypoxic tumour microenvironment is also a barrier for drug penetration, creating immunosuppressive environments, thus limiting treatment effectiveness [91]. Chemotherapy-induced hypoxia leads to the up-regulation of CXCL12/CXCR4 signalling, creating a vicious cycle that supports drug resistance and tumour regrowth [92]. Targeting CXCR4 in conjunction with conventional therapies may disrupt hypoxic signalling loops and restore therapeutic sensitivity, posing a potential strategy to overcome drug resistance in TNBC [93].

### 5.5. Facilitation of Tumour Cell Dormancy and Metastasis

The CXCL12/CXCR4 axis plays a significant role in tumour cell dormancy and metastasis in TNBC [58]. CXCR4 is commonly overexpressed in TNBC cells, while its ligand CXCL12 is preferentially secreted in distant organs, such as the lungs, liver, bone marrow, and lymph nodes, which are frequent metastatic sites for TNBC [94]. This chemokine distribution creates a trafficking gradient that facilitates the homing and migration of CXCR4-positive cancer cells to these metastatic niches. Once disseminated, a subpopulation of TNBC cells can enter a dormant state, characterised by cell cycle arrest and metabolic quiescence, rendering them insensitive to chemotherapy, which primarily targets actively dividing cells [95]. The CXCL12/CXCR4 axis may help maintain this dormant state through activation of downstream signalling pathways (e.g., PI3K/AKT, ERK1/2) and interactions with the tumour microenvironment, including stromal and immune cells, which support cell survival without promoting proliferation [57]. Dormant cells may persist in distant tissues for months or even years before reactivation under favourable conditions, eventually leading to metastatic outgrowth [96]. The CXCR4 axis further contributes to extravasation, angiogenesis, and immune evasion, all of which are crucial for the establishment of de novo metastatic colonisation [60]. Thus, therapeutic targeting of the CXCL12/CXCR4 axis may not only inhibit the initial seeding of metastasis but also late-stage recurrence and prolonged outcomes in patients with TNBC [97]. The CXCR4/CXCL12 axis is key in mediating drug resistance in TNBC via a multi-pronged mechanism of action, including pro-survival signalling, induction of EMT, maintenance of cancer stem cells, immune suppression, and metastatic dissemination [98]. Therapeutic repression of the CXCR4/CXCL12 axis via small molecule CXCR4 antagonists, monoclonal antibodies, or combination therapies with chemotherapy/immunotherapy options is highly promising to overcome resistance and improve clinical outcomes in patients with TNBC [29]. There is significant work to be conducted to identify and characterise interventions that optimise the therapeutic window and to identify patient populations who may benefit the most.

## 6. Strategies to Overcome CXCR4/CXCL12-Mediated Drug Resistance in TNBC

**Figure 5:**
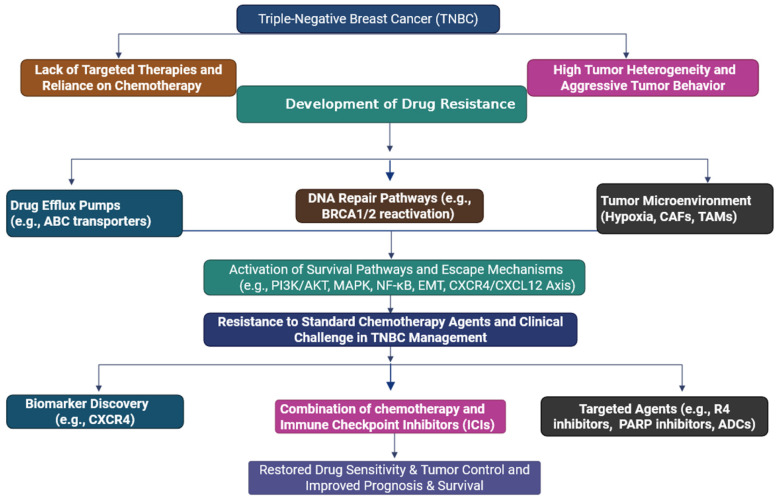
The figure depicts some of the major contributors to drug resistance in TNBC, which encompass a deficiency of targeted agents, increased tumour heterogeneity, the aggressive nature of the tumour, mechanisms of drug efflux, defective DNA repair processes, and externalities from the tumour microenvironment. These contribute to the activation of survival and escape pathways, further leading to resistance against conventional chemotherapy. Strategies to address this obstacle are biomarker discovery (e.g., CXCR4), combination therapies employing chemotherapy with ICIs, and targeted agents like PARP inhibitors and ADCs, with goals to restore drug sensitivity, enhance tumour control, and improve patient survival rates.

Addressing CXCR4/CXCL12-mediated drug resistance in TNBC necessitates strategies that target this chemokine axis and its effects, particularly through either direct blockade of the signalling pathways and cells involved or opening the door to further combinations [14]. The use of CXCR4 antagonists, such as plerixafor (AMD3100), balixafortide, and POL5551, is one such approach that, in tandem with standard chemotherapeutics (e.g., doxorubicin or paclitaxel), produced a synergistic effect, resensitizing TNBC cells to apoptosis, and reduced tumour burden [19]. Ultimately, plerixifor, balixafortide, and POL5551 antagonise CXCL12 binding and are helpful in inhibiting pro-survival PI3K/AKT and MAPK/ERK signalling pathways (Figure 5) [99]. A second developing approach to hierarchical blocking of the CXCR4 signalling axis in establishing reversal of immune evasion is the combinatorial use with immune checkpoint inhibitors [100]. Dual blockade strategies of immune checkpoint inhibitors and CXCR4 inhibitors will condition the tumour microenvironment, re-increase T cell infiltration, and improve therapeutic outcomes from immunotherapy (Table 3) [101]. Lastly, in a third developing approach in addressing CXCR4/CXCL12-mediated drug resistance in TNBC, pharmaceutical engineers are developing a nanoparticle-based delivery system to selectively direct the CXCR4 inhibitors and chemotherapeutics to the tumour tissue to limit systemic toxicity (Table 3) [14].

### 6.1. CXCR4 Antagonists

CXCR4 antagonists are a novel class of therapeutic agents that inhibit the CXCR4/CXCL12 signalling axis, which regulates tumour progression, metastasis, immune suppression, and chemoresistance by providing a microenvironment in which aggressive tumours like TNBC can thrive and evade the body’s defence mechanisms [13]. By inhibiting CXCR4 signalling, CXCR4 antagonists block downstream pathways, such as PI3K/AKT, MAPK/ERK, and JAK/STAT, that are responsible for cell survival signalling and chemoresistance in TNBC [108]. One of the more widely studied CXCR4 inhibitors is Plerixafor (AMD3100), which is FDA-approved for haematopoietic stem cell mobilisation in lymphoma and multiple myeloma [109]. Plerixafor has demonstrated the ability to inhibit metastases, decrease cancer stem cells, and sensitise tumours to chemotherapeutic agents, such as doxorubicin and paclitaxel, in preclinical models of TNBC [110]. The anti-metastatic effects of Plerixafor reported in TNBC preclinical models were due in part to inhibition of tumour cell homing to the CXCL12-rich environments, such as the bone marrow and lungs [111]. Another promising agent in development is Balixafortide (POL6326), a selective peptide antagonist of CXCR4 [112]. Balixafortide has moved to Phase I/II clinical trials for metastatic breast cancer, where it is being studied in combination with eribulin [102]. As a first-in-class agent, early data suggest improved response rates and tolerability in patients with metastatic breast cancer, highlighting the potential utility in advanced TNBC. Ulocuplumab (BMS-936564), a human monoclonal antibody against CXCR4, and MSX-122, a small molecule inhibitor with partial agonist properties that modulates tumour cell migration without altering normal haematopoiesis, are other investigational CXCR4 antagonists [113]. In summary, CXCR4 antagonists demonstrate the potential to combat therapeutic resistance through blocking tumour-stroma interactions, inhibiting cancer stem cell survival, and increasing immune infiltration. Clinical trials will continue to provide data to define the efficacy of CXCR4 antagonists, particularly when employed in rational combinations with chemotherapy, immune checkpoint inhibitors, or PARP inhibitors in TNBC [114].

### 6.2. Monoclonal Antibodies and Ligand Traps

Monoclonal antibodies (mAbs) and ligand traps are new therapeutic mechanisms to inhibit the CXCR4/CXCL12 axis, which promotes drug resistance in TNBC [14]. Biologics, such as mAbs and ligand traps, selectively target the receptor (CXCR4) or ligand (CXCL12) to inhibit a signalling loop that promotes tumour survival, immune suppression, and chemoresistance [22]. Ulocuplumab (BMS-936564) is a fully human monoclonal antibody that specifically binds to CXCR4 and inhibits the binding of CXCL12 in order to block ligand-induced receptor activation. Inhibiting CXCL12 action through ulocuplumab results in the inhibition of downstream pro-survival signals (PI3K/AKT, MAPK), reduction in maintenance of cancer stem cells, and increased chemotherapeutic-triggered apoptosis [22]. In preclinical models and Phase I/II studies, ulocuplumab has shown the ability to enhance tumour regression and immune cell infiltration alone or when combined with immune checkpoint inhibitors or cytotoxic components [97]. On the ligand-targeting side, NOX-A12 (Olaptesed pegol), a Spiegelmer-based ligand trap, binds to and neutralises CXCL12, thus preventing it from activating CXCR4 [115]. NOX-A12 has demonstrated effective anti-tumour activity by mobilising immune cells into the tumour microenvironment, disrupting the stromal protective benefits of tumour cells, and sensitising cancer cells to chemotherapy and radiotherapy. Early clinical data in solid tumours, including breast cancer, indicate that NOX-A12 is well tolerated and performs well in a combination regimen [103]. These biologics have several benefits that could be meaningfully helpful, including specificity, reduced off-target toxicity, and the potential to modulate the tumour microenvironment [116]. Together with conventional therapies or immuno-oncology therapies, monoclonal antibodies and ligand traps targeting the CXCR4/CXCL12 axis could alter the therapeutic resistance in TNBC, share tournament sustainability, and improve overall survival [29].

### 6.3. Combination Therapies

The CXCR4/CXCL12 pathway alone has shown promise in preclinical studies of TNBC [117]. The therapeutic effects of targeting this pathway are substantially enhanced when combined with other treatment modalities [117]. Combination therapies represent an emerging and rational means by which to counteract the complex involvement of CXCR4/CXCL12 in tumour survival and metastasis, immune escape, and drug resistance [14]. On the ligands-targeting front, NOX-A12 (Olaptesed pegol) is a Spiegelmer-based ligand trap that binds and neutralises CXCL12, effectively inhibiting CXCR4 activation [103]. NOX-A12 has demonstrated strong anti-tumour efficacy through mobilisation of immune cells into the tumour microenvironment, diminishing the tumour cells’ stroma-protection, and by sensitising the cancer cells to chemotherapy and radiotherapy. In early clinical studies involving solid tumours, including breast cancer, NOX-A12 has shown an acceptable tolerability profile and improvement in clinical outcomes in combination regimens [103]. Finally, biologics offer important advantages, including high specificity, low off-target toxicity, and modification of the tumour microenvironment. When combined with systemic therapies or immunotherapies, the use of monoclonal antibodies (mAbs) and ligand traps that target the CXCR4/CXCL12 pathway has the potential to overcome the development of therapeutic resistance in TNBC and improve long-term outcomes in patients [80].

### 6.4. CXCR4 Inhibitors with Chemotherapy

The combination of CXCR4 antagonists (such as plerixafor or balixafortide) with standard chemotherapeutics (e.g., paclitaxel, doxorubicin, or cisplatin) makes TNBC cells more sensitive to treatment by inhibiting stromal protection, decreasing the maintenance of cancer stem cells, and promoting apoptosis [24]. Platforms testing this combination therapy have yielded decreased tumour burden and metastasis in xenograft models [24].

### 6.5. CXCR4 Blockade with Immune Checkpoint Inhibitors (ICIs)

The immune-suppressing tumour microenvironment in TNBC typically limits the effectiveness of immune checkpoint inhibitors (ICIs) like anti-PD-1/PD-L1 [118]. CXCR4 inhibition can increase T cell infiltration while decreasing the populations of regulatory T cells (Tregs) and MDSCs. These changes promote the anti-tumour immune response [119]. Overall, early-phase trials combining CXCR4 inhibitors and ICIs have shown early promising immune activation in solid tumours that are refractory to previously unsuccessful lines of therapy [120].

### 6.6. CXCR4 Targeting with PARP Inhibitors

PARP inhibitors take advantage of DNA repair deficiencies in BRCA-mutated TNBC, but resistance often occurs [121]. Co-targeting CXCR4 may inhibit cancer stamens and hypoxia-induced survival signalling and further improve the efficacy of PARP inhibition [122].

### 6.7. Radiotherapy or Anti-Angiogenic Agents with CXCR4 Inhibition

CXCR4 blockade can normalise tumour vasculature and reduce hypoxia to sensitise tumours to radiation or a similar angiogenesis inhibitor (such as bevacizumab) [123]. As such, the blockade of CXCR4 in combination with other radiosensitising approaches can be ideal to enhance treatment response and reduce relapse in TNBC by targeting tumour cells and reconstructing the protective microenvironment mediated by CXCR4/CXCL12 signalling [15]. New nanotechnology platforms are under development to target the CXCR4/CXCL12 axis in TNBC [124]. A preclinical example used gold nanorods (AuNRs) conjugated to a CXCR4-peptide antagonist (E5). Invoking photothermal therapy with near-infrared laser irradiation activates the system to induce immunogenic cell death of TNBC cells [125]. The damaged tumour cell debris enhances antigen presentation and primes a robust anti-tumour immune response [126]. By concurrently disengaging the CXCR4/CXCL12-mediated immunosuppressive microenvironment and promoting tumour-specific immunity, this dual-action strategy provides a novel and synergistic approach to target drug resistance in triple-negative breast cancer [100].

### 6.8. Novel Nanotechnological

Novel nanotechnological platforms are exploring targeting of the CXCR4/CXCL12 axis in TNBC. One experimental model involves gold nanorods (AuNRs) conjugated to a CXCR4-peptide antagonist (E5) [125]. By adding near-infrared (NIR) laser irradiation to this system, photothermal therapy elicits immunogenic cell death of the TNBC cells [127]. The debris from the TNBC cells provides antigen presentation and promotes enhanced anti-tumour immunity within the microenvironment by resolving the CXCR4/CXCL12-mediated immunosuppressive microenvironment and promoting tumour-specific immunity [128]. This provides a new modus operandi and a novel synergistic means to exploit drug resistance in TNBC [104,105,106,107,129].

## 7. Clinical Trials to Overcome Drug Resistance in Triple-Negative Breast Cancer

CXCR4-targeted agents are moved through translational pathways, with balixafortide and leronlimab making initial progress in TNBC [129]. The future lies in exploration involving combinations, patient stratification, and immunomodulatory combinations [130]. X4P 001 is a selective oral CXCR4 antagonist that has been developed to block the CXCL12/CXCR4 signalling axis implicated in immune evasion, metastasis, and drug resistance [10]. A Phase I/II clinical trial is assessing the combination of X4P 001 with toripalimab (a PD-1 immune checkpoint inhibitor) in patients with unrespectable locally advanced or metastatic solid tumours, including TNBC [131]. This combination builds on the concept that the CXCR4 blockade may increase immune infiltration in the tumour microenvironment via reduction in T regulatory cells and myeloid-derived suppressor (MDSC) cells, thus promoting the activity of PD-1 inhibitors [132]. The study population consists of chemo-resistant malignancies, with a focus on patients who have failed standard lines of therapy [3]. The trial is designed to establish the safety, tolerability, pharmacokinetics, and early indication of efficacy, i.e., objective response rate (ORR) and progression-free survival (PFS), as well as correlation with biomarkers, including CXCR4 expression and immune modulation [133]. Preclinical studies have confirmed dual targeting of CXCR4/PD-1 can reverse immune suppression in TNBC models, therefore improving antitumour activity [97]. If successful, this trial will inform the development of a new immunotherapeutic strategy in the drug-resistant TNBC setting, potentially addressing one of the major clinical challenges in oncology (Table 4) [134]. POL5551 is a protein-epitope mimetic antagonist of CXCR4, designed to interfere with CXCL12–CXCR4 interactions involved in tumour metastasis and therapy resistance [135]. In preclinical orthotopic models of triple-negative breast cancer (TNBC), POL5551 significantly inhibited distant metastasis and, when given before and after eribulin, further reduced bone and liver tumour burden and prolonged survival relative to eribulin alone [135]. Based on the findings at that time, a Phase I clinical trial has begun to evaluate POL5551 in combination with eribulin in patients with relapsed or metastatic breast cancer, including TNBC [136]. The primary objectives are safety, maximum tolerated dose, and pharmacokinetics when administered with eribulin [136]. Secondary endpoints include exploratory efficacy endpoints, such as objective response rate and progression-free survival [137]. This clinical trial represents a significant shift in thinking about clinical therapy as a sequenced combination of therapies based on a “chemotherapy framing” strategy that has demonstrated the ability to sensitise tumour cells as they are mobilised away from protective microenvironments [138]. If the trial is successful, it may allow larger studies and possibly define a new therapy for reversing treatment resistance with drugs for advanced TNBC [139].

A novel preclinical approach, where 177Lu-labelled fibroblast activation protein (FAP)–targeted radiopharmaceutical was combined with the CXCR4 antagonist plerixafor (AMD3100) [30]. The radiolabelled combines selectively to cancer-associated fibroblasts (CAFs) that are plentiful in the tumour microenvironment, typically provide structural support, mediate immune suppression, and provide therapy resistance to established cancers [140]. Once bound, the radiopharmaceutical is internalised and subsequently releases targeted radiation, which disrupts CAFs and the tumour stroma. On the other hand, by binding to CXCR4 on tumour cells and stroma, plerixafor antagonises CXCR4 signalling, blockading chemokine-mediated immunosuppressive cell recruitment while augmenting immune infiltrates [11]. In concert with radiotherapy, the intended effects include physically disrupting the tumour-supported scaffold, releasing immune suppression, improving radio sensitivity, and enhancing anti-tumour immune responses [141].

Preclinical in vivo research in murine TNBC model systems has revealed a decrease in stromal density and a major increase in infiltration of cytotoxic T cells and tumour regression in comparison to either modality alone [142]. These preliminary results suggest that the use of stromal ablation in combination with immunomodulation is a rational potential strategy to get ahead of stromal marrow-mediated drug resistance and could pave the way for potential applications in translational medicine down the road [143]. Now, one promising preclinical strategy is to combine radionuclide therapy with plerixafor (AMD3100), a frequently used antagonist of CXCR4 [144]. The rationale behind this approach is to first simultaneously deplete cancer-associated fibroblasts (CAFs), which are a major aspect of the tumour stroma, while inhibiting CXCR4-mediated immune suppression [145,146,147,148]. The intention of targeted stroma ablation, while enhancing effective immune cell infiltration, is to ultimately circumvent the resistance mechanisms, particularly in immunologically cold and therapy-refractory models of tumours like TNBC [120]. Notably, the approach has been shown to significantly enhance T-cell activation and tumour regression in preclinical models, which suggests it has translational possibilities in TNBC and potentially other refractory solid tumours going forward.

**Table 4:** Clinical Trials to Overcome Drug Resistance in Triple-Negative Breast Cancer.

S. No.	Trial/Agent	Phase	Combination	Target Population	Key Objectives	Status	References
1	CTCE-9908	Phase I	Peptide-based CXCL12 inhibitor	Solid tumours, including breast cancer	Safety and pharmacokinetics	Completed (no TNBC-specific results published)	[148]
2	POL5551 + Eribulin	Phase I	CXCR4 antagonist + chemotherapy	Advanced breast cancer	Dose escalation, safety, preliminary anti-tumour activity	Initiated	[135]
3	Balixafortide (POL6326) + Eribulin	Phase III	CXCR4 antagonist + chemotherapy	HER2-negative MBC, including TNBC	Evaluate efficacy and safety vs. eribulin alone (PFS, OS)	Ongoing (FORTRESS Trial, NCT03786094)	[102]
4	NOX-A12 (Olaptesed Pegol)	Phase I/II	CXCL12 neutralisation + checkpoint inhibitor	Solid tumours and hematological cancers	Blockade of tumour-stroma interaction, immune enhancement	Completed (other cancers)	[103]
5	BL-8040 (Motixafortide)	Phase II	CXCR4 inhibitor + pembrolizumab	Pancreatic and other solid tumours	Evaluate immune activation and anti-tumour activity	Completed in other cancers, potential for TNBC	[147]
6	X4P-001 + Toripalimab	Phase I/II	CXCR4 inhibitor + PD-1 inhibitor	Solid tumours, including TNBC	Assess safety, tolerability, and preliminary efficacy	Ongoing	[146]
7	Plerixafor (AMD3100) + Radiotherapy	Preclinical	CXCR4 inhibitor + FAP-targeted radionuclide	TNBC and CAF-rich tumours	Deplete CAFs, enhance T-cell infiltration	Preclinical	[144]

## 8. Limitations of CXCR4/CXCL12-Targeted Therapy in TNBC

Although preclinical data supported a strong rationale for CXCR4/CXCL12 as a therapeutic target, the process of translating this target into the clinic for the treatment of TNBC is fraught with issues [149,150]. The most significant issue is tumour heterogeneity, as TNBC is a diverse disease with many molecular subtypes that exhibit variable levels of CXCR4 expression and reliance on CXCR4-mediated signalling [151]. Therefore, this heterogeneity is one contributor to differential therapeutic responsiveness in clinically diverse TNBC populations, and it makes it challenging to develop consensus patient stratification and matching processes in clinical trials [152]. These factors underscore the problem of non-issue consistency across patient populations if a uniform approach is employed toward targeting CXCR4 [151]. Systemic toxicity is another significant limitation because CXCR4 has uniquely important roles in several important normal physiological processes, such as hematopoiesis, immune cell trafficking, and organ development. There is a risk that pharmacologic inhibition of CXCR4 could disrupt immune homeostasis and mobilise hematopoietic stem cells (HSCs) from the bone marrow niche with a subsequent increase in the risk of infection or ectopic off-target organ effects [150]. The translational challenge in regard to effective blockade of CXCR4 in tumours while minimising both systemic adverse events will be key for the future development of CXCR4 blockade therapies [149]. While targeting the CXCR4/CXCL12 axis holds considerable promise in overcoming drug resistance in TNBC, several limitations hinder its clinical translation and long-term efficacy [149]. These challenges include biological complexity, compensatory resistance mechanisms, off-target effects, and delivery issues, all of which must be addressed to realise its full therapeutic potential [120].

### 8.1. Lack of Predictive Biomarkers

The efficacy of targeted therapy depends heavily on identifying biomarkers that predict therapeutic response [150]. However, no validated biomarker currently exists to select TNBC patients who would most benefit from CXCR4 inhibition. While high CXCR4 expression is associated with poor prognosis and enhanced chemoresistance, it does not reliably predict drug sensitivity [151]. Moreover, a lack of predictive biomarkers is a serious limitation to clinical application. To date, there are no validated biomarkers that can reliably identify which patients will benefit from CXCR4-targeted therapies or monitor therapeutic efficacy [150]. Clinical trials could potentially worsen the situation of heterogeneous patient populations that could dilute measurable benefit and limit regulatory approval.

### 8.2. Drug Delivery and Pharmacokinetics

Most CXCR4 inhibitors, such as peptide-based agents (e.g., balixafortide) and monoclonal antibodies (e.g., ulocuplumab), suffer from poor oral bioavailability, short half-life, and delivery challenges [113]. These issues necessitate frequent dosing and increase treatment burden. Effective tumour-specific delivery remains especially challenging in fibrotic and hypoxic TNBC environments.

### 8.3. Off-Target Effects and Toxicity

CXCR4 is also expressed in normal tissues, such as hematopoietic stem cells, immune cells, and endothelial cells. Systemic inhibition may lead to leukocytosis, immune dysregulation, and impaired tissue regeneration, which may limit dosage and duration—particularly in combination regimens [26].

### 8.4. Tumour Heterogeneity and Plasticity

TNBC is highly heterogeneous at the genetic and phenotypic levels. Variability in CXCR4 expression and CXCL12 dependency among subclones leads to inconsistent therapeutic responses [152]. Furthermore, cancer cells may adopt alternative survival pathways or phenotypes that bypass CXCR4 inhibition.

### 8.5. Incomplete Clinical Validation

Despite promising preclinical outcomes, clinical validation remains limited. Most trials of CXCR4 antagonists are in early phases with small patient cohorts, preventing conclusive evaluation of long-term efficacy. Well-designed, large-scale, biomarker-guided trials are urgently needed [152].

In order to move towards clinical application, future studies need to focus on identifying TNBC sub-groups with CXCR4 dependency, developing predictive biomarkers, and designing combination strategies with optimal efficacy and less toxicity [149]. Overcoming these obstacles is necessary for the CXCR4/CXCL12 axis to demonstrate its full therapeutic ability to overcome drug resistance and ultimately result in improved outcomes for patients with TNBC.

## 9. Future Directions for Targeting the CXCR4/CXCL12 Axis in TNBC

Despite current limitations, the future of CXCR4/CXCL12-targeted therapy lies in multimodal and personalised strategies. Identifying patients with elevated CXCR4 expression or increased CXCL12 secretion may enable stratified therapy [153]. Liquid biopsies and CXCR4-targeted imaging could provide real-time assessment for therapy selection [154]. Development of novel CXCR4 inhibitors with improved oral bioavailability, pharmacokinetics, and reduced toxicity will be critical [155]. Dual-targeting agents that simultaneously block CXCR4 and other pathways (e.g., EGFR, PD-1, and VEGF) may overcome compensatory resistance mechanisms [105]. CXCR4 blockade has been shown to increase immune cell infiltration in tumours. Combining CXCR4 antagonists with immune checkpoint inhibitors (e.g., anti-PD-1/PD-L1), cancer vaccines, or adoptive cell therapy holds the potential to convert immunologically “cold” TNBC tumours into “hot” responsive ones. Nanoparticles capable of co-delivering CXCR4 inhibitors with chemotherapeutics or RNA-based drugs can enhance efficacy and reduce systemic toxicity [156]. Cancer-specific and stimulus-responsive nanocarriers could achieve precise drug release in CXCL12-rich environments [157]. As the CXCL12/CXCR4 axis interacts closely with stromal and immune cells, targeting these interactions, possibly with agents like TGF-β inhibitors or myeloid-targeting therapies, may disrupt resistance-supporting niches. Rigorous clinical validation through biomarker-enhanced, multi-arm trials is essential [158,159,160,161,162]. Collaboration among academia, industry, and regulatory bodies will be key to translating preclinical promise into approved therapies.

## 10. Conclusions

The CXCR4/CXCL12 axis occupies the intersection of intrinsic tumour cell survival, stemness, metastatic dissemination, and immune suppression. Its targeted therapy presents a multi-faceted assault on key resistance mechanisms in TNBC. Although single-agent therapy is unlikely to be sufficient, rational combinations, enhanced by accurate delivery, biomarker stratification, and microenvironmental reconditioning, are a compelling therapeutic vision. The future direction demands concerted action between oncologists, immunologists, biomaterials scientists, and clinical researchers. Nanotechnology, real-time monitoring of biomarkers and adaptive design will be cornerstones. The goal is as follows: translate CXCR4-targeted approaches from a hope into a revolutionary, clinically proven means of overcoming drug resistance and enhancing outcomes in TNBC. In conclusion, inhibition of the CXCR4/CXCL12 axis is a beacon of hope to defeat drug-resistant TNBC. With ongoing innovation combining molecular targeting, immunotherapy, and advanced delivery systems, an era in which resistant TNBC is a treatable and even curable disease comes closer into sight.

## Figures and Tables

**Table 1:** Triple-negative breast cancer poses a significant clinical challenge due to its high rate of drug resistance.

S. No.	Characteristic	Particulars	Reference
1	Subtype Characteristics	Lacks ER, PR, and HER2 expression; highly aggressive; limited targeted therapies.	[39]
2	Emerging Resistance Pathways	PI3K/AKT/mTOR, MAPK/ERK, JAK/STAT, NF-κB.	[43]
3	Tumour Microenvironment	Hypoxia, tumour-associated macrophages (TAMs), cancer-associated fibroblasts (CAFs), cytokine signalling (e.g., CXCR4/CXCL12).	[42]
4	Clinical Consequences	Poor prognosis, high recurrence, limited response to standard therapies.	[44]
5	Major Mechanisms of Resistance	Genetic mutations: TP53, BRCA1/2, PIK3CA, PTEN Epigenetic changes: Histone modifications, DNA methylation EMT and stemness: Enhanced invasion and self-renewal Drug efflux: ABC transporters (P-gp, BCRP) Immune evasion: Downregulation of MHC-I, T-cell dysfunction	[41]
6	Primary Treatment	Cytotoxic chemotherapy (e.g., doxorubicin, paclitaxel, carboplatin).	[40]
7	Strategies to Overcome Resistance	PARP inhibitors (e.g., olaparib in BRCA-mutated TNBC) CXCR4 antagonists (e.g., AMD3100) Immunotherapy (e.g., checkpoint inhibitors) Combination therapies targeting signalling and immune pathways	[45]

**Table 2:** Mechanism of CXCR4/CXCL12 Axis in Drug Resistance in Triple-Negative Breast Cancer.

S. No.	Mechanism	Description	Downstream Pathways/Effects	Impact on Drug Resistance	Reference
1	Epithelial–Mesenchymal Transition (EMT)	CXCR4 signalling upregulates EMT transcription factors, increasing cell plasticity and invasiveness.	Snail, Slug, Twist, ZEB1	Enhances migratory capacity, reduces drug sensitivity.	[61]
2	Metastatic Niche Formation	CXCR4^+^ cells home to distant CXCL12-rich organs (e.g., lung, liver, bone) and enter dormancy.	CXCL12 gradients, integrins	Enables survival during systemic therapy and leads to late recurrence.	[10]
3	Cancer Stem Cell (CSC) Maintenance	Supports self-renewal and pluripotency of CSCs that are inherently resistant to therapy.	Oct4, Sox2, Nanog, Wnt/β-catenin	Sustains tumour-initiating, drug-resistant cell populations.	[62]
4	Pro-survival Signalling Activation	CXCL12 binding to CXCR4 activates anti-apoptotic signalling cascades.	PI3K/AKT, MAPK/ERK, NF-κB	Inhibits apoptosis, promotes cell survival under chemotherapeutic stress.	[57]
5	Hypoxia and Stromal Protection	Hypoxic niches with high CXCL12 expression protect tumour cells via paracrine signalling and reduced vascular access.	HIF-1α, TGF-β	Limits drug penetration; maintains resistant cell niches.	[63]
6	Immune Suppression	Recruits regulatory T cells (Tregs), MDSCs, and excludes CD8^+^ T cells from tumour site.	CXCR4-dependent immune exclusion	Reduces efficacy of immune-based therapies (e.g., checkpoint inhibitors).	[63]

**Table 3:** Strategies to Overcome CXCR4/CXCL12-Mediated Drug Resistance in TNBC.

S. No.	Description	Examples/Agents	Mechanism of Action	Clinical Status	References
1	Direct inhibition of CXCR4 receptor to block CXCL12-mediated survival and migration signalling.	Plerixafor (AMD3100), Balixafortide, POL5551	Disrupts CXCL12/CXCR4 binding; inhibits EMT, CSC maintenance, and stromal protection	Plerixafor approved (other agents in trials)	[19]
2	Co-administration of CXCR4 inhibitors with standard chemotherapeutics.	Balixafortide + Eribulin, Plerixafor + Paclitaxel	Enhances cytotoxic efficacy by sensitising resistant cancer cells	Ongoing clinical trials (e.g., FORTRESS)	[102]
3	Binds or depletes CXCL12 to prevent receptor activation.	NOX-A12 (olaptesed pegol), CTCE-9908	Reduces stromal-mediated resistance and immune evasion	Early-phase trials/preclinical	[103]
4	Inhibits both CXCR4 and CXCR7 receptors to overcome pathway redundancy.	Dual antagonists in development	Prevents bypass signalling and reinforces blockade of chemokine activity	Preclinical stage	[28]
5	Plant-derived agents that downregulate CXCR4 or associated pathways.	Z-Guggulsterone, Curcumin	Inhibits NF-κB/CXCR4 signalling; reduces metastasis and stemness	Preclinical	[104]
6	Targeted delivery of CXCR4 antagonists and chemotherapeutics to the tumour microenvironment.	CXCR4-targeted liposomes, AuNR-E5 nanocarriers	Enhances drug accumulation in tumour tissue; improves precision and reduces toxicity	Preclinical/experimental	[105]
7	Augments response to immune checkpoint inhibitors by improving immune infiltration.	Plerixafor + anti-PD-1 (Toripalimab), BL-8040 combos	Reverses immune exclusion; enhances CD8^+^ T cell infiltration	Phase I/II trials	[106]
8	Combines stromal ablation with CXCR4 inhibition to disrupt the protective tumour niche.	177Lu-FAPi + AMD3100	Depletes CAFs and enhances immune response within TME	Preclinical	[30]
9	Inhibition of signalling pathways downstream of CXCR4 (e.g., PI3K/AKT, STAT3).	PI3K inhibitors, JAK/STAT blockers	Prevents survival signalling even if CXCR4 is partially active	Clinical and preclinical stages	[107]

## Data Availability

No new data were created or analysed in this study. Data sharing is not applicable to this article.

## Abbreviations

TNBC	Triple-Negative Breast Cancer
CXCR4	C-X-C Chemokine Receptor Type 4
CXCL12	C-X-C Motif Chemokine Ligand 12
SDF-1	Stromal-Derived Factor-1
ER	Estrogen Receptor
PR	Progesterone Receptor
HER2	Human Epidermal Growth Factor Receptor 2
GPCR	G-Protein-Coupled Receptor
TME	Tumour Microenvironment
EMT	Epithelial–Mesenchymal Transition
CSC	Cancer Stem Cell
ABC	ATP-Binding Cassette
PI3K	Phosphoinositide 3-Kinase
AKT	Protein Kinase B
MAPK/ERK	Mitogen-Activated Protein Kinase/Extracellular Signal-Regulated Kinase
JAK/STAT	Janus Kinase/Signal Transducers and Activators of Transcription
NF-κB	Nuclear Factor Kappa-light-chain-enhancer of Activated B Cells
MHC-I	Major Histocompatibility Complex Class I
Tregs	Regulatory T Cells
MDSCs	Myeloid-Derived Suppressor Cells
TAMs	Tumour-Associated Macrophages
ICIs	Immune Checkpoint Inhibitors
PD-1	Programmed Cell Death Protein 1
PD-L1	Programmed Death-Ligand 1
HIF-1α	Hypoxia-Inducible Factor-1 Alpha
BRCA1/2	Breast Cancer Gene 1/2
TP53	Tumour Protein p53
PIK3CA	Phosphatidylinositol-4,5-Bisphosphate 3-Kinase Catalytic Subunit Alpha
PTEN	Phosphatase and Tensin Homolog
AR	Androgen Receptor
BCRP	Breast Cancer Resistance Protein
P-gp	P-glycoprotein
RTKs	Receptor Tyrosine Kinases
EGFR	Epidermal Growth Factor Receptor
FGFR1/2	Fibroblast Growth Factor Receptor 1/2
VEGFR	Vascular Endothelial Growth Factor Receptor
PARP	Poly (ADP-ribose) Polymerase
ALDH1	Aldehyde Dehydrogenase 1
CTL	Cytotoxic T Lymphocyte
FAP	Fibroblast Activation Protein
AuNRs	Gold Nanorods
NIR	Near Infrared
mAbs	Monoclonal Antibodies
OS	Overall Survival
PFS	Progression-Free Survival
ORR	Objective Response Rate

## References

[1] Zagami P., Carey L.A. (2022). Triple Negative Breast Cancer: Pitfalls and Progress. npj Breast Cancer.

[2] Yao H., He G., Yan S., Chen C., Song L., Rosol T.J., Deng X. (2017). Triple-Negative Breast Cancer: Is There a Treatment on the Horizon?. Oncotarget.

[3] Obidiro O., Battogtokh G., Akala E.O. (2023). Triple Negative Breast Cancer Treatment Options and Limitations: Future Outlook. Pharmaceutics.

[4] Bhol C.S., Panigrahi D.P., Praharaj P.P., Mahapatra K.K., Patra S., Mishra S.R., Behera B.P., Bhutia S.K. (2020). Epigenetic Modifications of Autophagy in Cancer and Cancer Therapeutics. Semin. Cancer Biol..

[5] Xie Y., Wang X., Wang W., Pu N., Liu L. (2025). Epithelial-Mesenchymal Transition Orchestrates Tumor Microenvironment: Current Perceptions and Challenges. J. Transl. Med..

[6] Choi Y., Yu A.-M. (2014). ABC Transporters in Multidrug Resistance and Pharmacokinetics, and Strategies for Drug Development. Curr. Pharm. Des..

[7] Xiong N., Wu H., Yu Z. (2024). Advancements and Challenges in Triple-Negative Breast Cancer: A Comprehensive Review of Therapeutic and Diagnostic Strategies. Front. Oncol..

[8] Bai X., Ni J., Beretov J., Graham P., Li Y. (2021). Triple-Negative Breast Cancer Therapeutic Resistance: Where Is the Achilles’ Heel?. Cancer Lett..

[9] Sabit H., Adel A., Abdelfattah M.M., Ramadan R.M., Nazih M., Abdel-Ghany S., El-hashash A., Arneth B. (2025). The Role of Tumor Microenvironment and Immune Cell Crosstalk in Triple-Negative Breast Cancer (TNBC): Emerging Therapeutic Opportunities. Cancer Lett..

[10] Shi Y., Riese D.J., Shen J. (2020). The Role of the CXCL12/CXCR4/CXCR7 Chemokine Axis in Cancer. Front. Pharmacol..

[11] Rueda A., Serna N., Mangues R., Villaverde A., Unzueta U. (2025). Targeting the Chemokine Receptor CXCR4 for Cancer Therapies. Biomark. Res..

[12] Mortezaee K. (2020). CXCL12/CXCR4 Axis in the Microenvironment of Solid Tumors: A Critical Mediator of Metastasis. Life Sci..

[13] Yang Y., Li J., Lei W., Wang H., Ni Y., Liu Y., Yan H., Tian Y., Wang Z., Yang Z. (2023). CXCL12-CXCR4/CXCR7 Axis in Cancer: From Mechanisms to Clinical Applications. Int. J. Biol. Sci..

[14] Khaleel A.Q., Altalbawy F.M.A., Jabir M.S., Hasan T.F., Jain V., Abbot V., Nakash P., Kumar M.R., Mustafa Y.F., Jawad M.A. (2025). CXCR4/CXCL12 Blockade Therapy; a New Horizon in TNBC Therapy. Med. Oncol..

[15] Liang S., Peng X., Li X., Yang P., Xie L., Li Y., Du C., Zhang G. (2015). Silencing of CXCR4 Sensitizes Triple-Negative Breast Cancer Cells to Cisplatin. Oncotarget.

[16] Guo Z., Han S. (2023). Targeting Cancer Stem Cell Plasticity in Triple-Negative Breast Cancer. Explor. Target. Anti-Tumor Ther..

[17] Huang Y., Hong W., Wei X. (2022). The Molecular Mechanisms and Therapeutic Strategies of EMT in Tumor Progression and Metastasis. J. Hematol. Oncol..

[18] Wang Y., Xie Y., Oupický D. (2016). Potential of CXCR4/CXCL12 Chemokine Axis in Cancer Drug Delivery. Curr. Pharmacol. Rep..

[19] De Clercq E. (2010). Recent Advances on the Use of the CXCR4 Antagonist Plerixafor (AMD3100, Mozobil^TM^) and Potential of Other CXCR4 Antagonists as Stem Cell Mobilizers. Pharmacol. Ther..

[20] Li Z., Lu H., Zhang Y., Lv J., Zhang Y., Xu T., Yang D., Duan Z., Guan Y., Jiang Z. (2024). Blocking CXCR4-CARM1-YAP Axis Overcomes Osteosarcoma Doxorubicin Resistance by Suppressing Aerobic Glycolysis. Cancer Sci..

[21] Mezzapelle R., Leo M., Caprioglio F., Colley L.S., Lamarca A., Sabatino L., Colantuoni V., Crippa M.P., Bianchi M.E. (2022). CXCR4/CXCL12 Activities in the Tumor Microenvironment and Implications for Tumor Immunotherapy. Cancers.

[22] Zhou W., Guo S., Liu M., Burow M.E., Wang G. (2019). Targeting CXCL12/CXCR4 Axis in Tumor Immunotherapy. Curr. Med. Chem..

[23] Romain B., Hachet-Haas M., Rohr S., Brigand C., Galzi J.-L., Gaub M.-P., Pencreach E., Guenot D. (2014). Hypoxia Differentially Regulated CXCR4 and CXCR7 Signaling in Colon Cancer. Mol. Cancer.

[24] Lefort S., Thuleau A., Kieffer Y., Sirven P., Bieche I., Marangoni E., Vincent-Salomon A., Mechta-Grigoriou F. (2017). CXCR4 Inhibitors Could Benefit to HER2 but Not to Triple-Negative Breast Cancer Patients. Oncogene.

[25] Pradhan R., Dey A., Taliyan R., Puri A., Kharavtekar S., Dubey S.K. (2023). Recent Advances in Targeted Nanocarriers for the Management of Triple Negative Breast Cancer. Pharmaceutics.

[26] Gupta N., Mohan C.D., Shanmugam M.K., Jung Y.Y., Chinnathambi A., Alharbi S.A., Ashrafizadeh M., Mahale M., Bender A., Kumar A.P. (2023). CXCR4 Expression Is Elevated in TNBC Patient Derived Samples and Z-Guggulsterone Abrogates Tumor Progression by Targeting CXCL12/CXCR4 Signaling Axis in Preclinical Breast Cancer Model. Environ. Res..

[27] El Hejjioui B., Lamrabet S., Amrani Joutei S., Senhaji N., Bouhafa T., Malhouf M.A., Bennis S., Bouguenouch L. (2023). New Biomarkers and Treatment Advances in Triple-Negative Breast Cancer. Diagnostics.

[28] Santagata S., Ieranò C., Trotta A.M., Capiluongo A., Auletta F., Guardascione G., Scala S. (2021). CXCR4 and CXCR7 Signaling Pathways: A Focus on the Cross-Talk Between Cancer Cells and Tumor Microenvironment. Front. Oncol..

[29] Smaldone G., Di Matteo F., Castelluccio R., Napolitano V., Miranda M.R., Manfra M., Campiglia P., Vestuto V. (2025). Targeting the CXCR4/CXCL12 Axis in Cancer Therapy: Analysis of Recent Advances in the Development of Potential Anticancer Agents. Molecules.

[30] Bao G., Wang Z., Liu L., Zhang B., Song S., Wang D., Cheng S., Moon E.-S., Roesch F., Zhao J. (2024). Targeting CXCR4/CXCL12 Axis via [^177^Lu]Lu-DOTAGA.(SA.FAPi)_2_ with CXCR4 Antagonist in Triple-Negative Breast Cancer. Eur. J. Nucl. Med. Mol. Imaging.

[31] Toft D.J., Cryns V.L. (2011). Minireview: Basal-Like Breast Cancer: From Molecular Profiles to Targeted Therapies. Mol. Endocrinol..

[32] Sporikova Z., Koudelakova V., Trojanec R., Hajduch M. (2018). Genetic Markers in Triple-Negative Breast Cancer. Clin. Breast Cancer.

[33] Soung Y.H., Ju J., Chung J. (2023). The Sensitization of Triple-Negative Breast Cancers to Poly ADP Ribose Polymerase Inhibition Independent of BRCA1/2 Mutation Status by Chemically Modified microRNA-489. Cells.

[34] Marvalim C., Datta A., Lee S.C. (2023). Role of P53 in Breast Cancer Progression: An Insight into P53 Targeted Therapy. Theranostics.

[35] Garg P., Ramisetty S., Nair M., Kulkarni P., Horne D., Salgia R., Singhal S.S. (2025). Strategic Advancements in Targeting the PI3K/AKT/mTOR Pathway for Breast Cancer Therapy. Biochem. Pharmacol..

[36] Chatterjee S., Behnam Azad B., Nimmagadda S. (2014). The Intricate Role of CXCR4 in Cancer. Advances in Cancer Research.

[37] Lei Z., Tian Q., Teng Q., Wurpel J.N.D., Zeng L., Pan Y., Chen Z. (2023). Understanding and Targeting Resistance Mechanisms in Cancer. MedComm.

[38] Lee J. (2023). Current Treatment Landscape for Early Triple-Negative Breast Cancer (TNBC). J. Clin. Med..

[39] Onitilo A.A., Engel J.M., Greenlee R.T., Mukesh B.N. (2009). Breast Cancer Subtypes Based on ER/PR and Her2 Expression: Comparison of Clinicopathologic Features and Survival. Clin. Med. Res..

[40] Skverchinskaya E., Levdarovich N., Ivanov A., Mindukshev I., Bukatin A. (2023). Anticancer Drugs Paclitaxel, Carboplatin, Doxorubicin, and Cyclophosphamide Alter the Biophysical Characteristics of Red Blood Cells, In Vitro. Biology.

[41] Ouedraogo S.Y., Zoure A.A., Zeye M.M.J., Kiendrebeogo T.I., Zhou X., Sawadogo A.Y., Simpore J., Chen H. (2022). BRCA1, BRCA2, TP53, PIK3CA, PTEN and AKT1 Genes Mutations in Burkina Faso Breast Cancer Patients: Prevalence, Spectrum and Novel Variant. Mol. Genet. Genom..

[42] Gunaydin G. (2021). CAFs Interacting With TAMs in Tumor Microenvironment to Enhance Tumorigenesis and Immune Evasion. Front. Oncol..

[43] Steelman L.S., Chappell W.H., Abrams S.L., Kempf C.R., Long J., Laidler P., Mijatovic S., Maksimovic-Ivanic D., Stivala F., Mazzarino M.C. (2011). Roles of the Raf/MEK/ERK and PI3K/PTEN/Akt/mTOR Pathways in Controlling Growth and Sensitivity to Therapy-Implications for Cancer and Aging. Aging.

[44] Stefani C., Miricescu D., Stanescu-Spinu I.-I., Nica R.I., Greabu M., Totan A.R., Jinga M. (2021). Growth Factors, PI3K/AKT/mTOR and MAPK Signaling Pathways in Colorectal Cancer Pathogenesis: Where Are We Now?. Int. J. Mol. Sci..

[45] Zou Y., Zhang H., Chen P., Tang J., Yang S., Nicot C., Guan Z., Li X., Tang H. (2025). Clinical Approaches to Overcome PARP Inhibitor Resistance. Mol. Cancer.

[46] Błaszczak E., Miziak P., Odrzywolski A., Baran M., Gumbarewicz E., Stepulak A. (2025). Triple-Negative Breast Cancer Progression and Drug Resistance in the Context of Epithelial–Mesenchymal Transition. Cancers.

[47] Haque M., Shyanti R.K., Mishra M.K. (2024). Targeted Therapy Approaches for Epithelial-Mesenchymal Transition in Triple Negative Breast Cancer. Front. Oncol..

[48] Nedeljković M., Damjanović A. (2019). Mechanisms of Chemotherapy Resistance in Triple-Negative Breast Cancer—How We Can Rise to the Challenge. Cells.

[49] Park S.-Y., Choi J.-H., Nam J.-S. (2019). Targeting Cancer Stem Cells in Triple-Negative Breast Cancer. Cancers.

[50] Kumar H., Gupta N.V., Jain R., Madhunapantula S.V., Babu C.S., Kesharwani S.S., Dey S., Jain V. (2023). A Review of Biological Targets and Therapeutic Approaches in the Management of Triple-Negative Breast Cancer. J. Adv. Res..

[51] Sharma P., Barlow W.E., Godwin A.K., Pathak H., Isakova K., Williams D., Timms K.M., Hartman A.R., Wenstrup R.J., Linden H.M. (2018). Impact of Homologous Recombination Deficiency Biomarkers on Outcomes in Patients with Triple-Negative Breast Cancer Treated with Adjuvant Doxorubicin and Cyclophosphamide (SWOG S9313). Ann. Oncol..

[52] Rose M., Burgess J.T., O’Byrne K., Richard D.J., Bolderson E. (2020). PARP Inhibitors: Clinical Relevance, Mechanisms of Action and Tumor Resistance. Front. Cell Dev. Biol..

[53] Goenka A., Khan F., Verma B., Sinha P., Dmello C.C., Jogalekar M.P., Gangadaran P., Ahn B. (2023). Tumor Microenvironment Signaling and Therapeutics in Cancer Progression. Cancer Commun..

[54] Ponnusamy L., Mahalingaiah P.K.S., Chang Y.-W., Singh K.P. (2019). Role of Cellular Reprogramming and Epigenetic Dysregulation in Acquired Chemoresistance in Breast Cancer. Cancer Drug Resist..

[55] Shi Z.-D., Pang K., Wu Z.-X., Dong Y., Hao L., Qin J.-X., Wang W., Chen Z.-S., Han C.-H. (2023). Tumor Cell Plasticity in Targeted Therapy-Induced Resistance: Mechanisms and New Strategies. Signal Transduct. Target. Ther..

[56] Nguyen K.T.P., Druhan L.J., Avalos B.R., Zhai L., Rauova L., Nesmelova I.V., Dréau D. (2020). CXCL12-CXCL4 Heterodimerization Prevents CXCL12-Driven Breast Cancer Cell Migration. Cell. Signal..

[57] Garg P., Jallepalli V.R., Verma S. (2024). Unravelling the CXCL12/CXCR4 Axis in Breast Cancer: Insights into Metastasis, Microenvironment Interactions, and Therapeutic Opportunities. Hum. Gene.

[58] Yang P., Hu Y., Zhou Q. (2020). The CXCL12-CXCR4 Signaling Axis Plays a Key Role in Cancer Metastasis and Is a Potential Target for Developing Novel Therapeutics against Metastatic Cancer. Curr. Med. Chem..

[59] Zielińska K.A., Katanaev V.L. (2020). The Signaling Duo CXCL12 and CXCR4: Chemokine Fuel for Breast Cancer Tumorigenesis. Cancers.

[60] Anastasiadou D.P., Quesnel A., Duran C.L., Filippou P.S., Karagiannis G.S. (2024). An Emerging Paradigm of CXCL12 Involvement in the Metastatic Cascade. Cytokine Growth Factor Rev..

[61] Jung Y.-D., Shim J.-W., Park S.-J., Choi S.H., Yang K., Heo K., Park M.-T. (2015). Downregulation of UHRF1 Promotes EMT via Inducing CXCR4 in Human Cancer Cells. Int. J. Oncol..

[62] Makena M.R., Ranjan A., Thirumala V., Reddy A.P. (2020). Cancer Stem Cells: Road to Therapeutic Resistance and Strategies to Overcome Resistance. Biochim. Biophys. Acta (BBA)-Mol. Basis Dis..

[63] Zhi S., Chen C., Huang H., Zhang Z., Zeng F., Zhang S. (2024). Hypoxia-Inducible Factor in Breast Cancer: Role and Target for Breast Cancer Treatment. Front. Immunol..

[64] Kiss R.C., Xia F., Acklin S. (2021). Targeting DNA Damage Response and Repair to Enhance Therapeutic Index in Cisplatin-Based Cancer Treatment. Int. J. Mol. Sci..

[65] Zhang H., Yue X., Chen Z., Liu C., Wu W., Zhang N., Liu Z., Yang L., Jiang Q., Cheng Q. (2023). Define Cancer-Associated Fibroblasts (CAFs) in the Tumor Microenvironment: New Opportunities in Cancer Immunotherapy and Advances in Clinical Trials. Mol. Cancer.

[66] Mollaei M., Hassan Z.M., Khorshidi F., Langroudi L. (2021). Chemotherapeutic Drugs: Cell Death- and Resistance-Related Signaling Pathways. Are They Really as Smart as the Tumor Cells?. Transl. Oncol..

[67] Yen J.-H., Chang C.-C., Hsu H.-J., Yang C.-H., Mani H., Liou J.-W. (2024). C-X-C Motif Chemokine Ligand 12―C-X-C Chemokine Receptor Type 4 Signaling Axis in Cancer and the Development of Chemotherapeutic Molecules. Tzu Chi Med. J..

[68] He Y., Sun M.M., Zhang G.G., Yang J., Chen K.S., Xu W.W., Li B. (2021). Targeting PI3K/Akt Signal Transduction for Cancer Therapy. Signal Transduct. Target. Ther..

[69] Hu X., Li J., Fu M., Zhao X., Wang W. (2021). The JAK/STAT Signaling Pathway: From Bench to Clinic. Signal Transduct. Target. Ther..

[70] Kim D., Xing T., Yang Z., Dudek R., Lu Q., Chen Y.-H. (2017). Epithelial Mesenchymal Transition in Embryonic Development, Tissue Repair and Cancer: A Comprehensive Overview. J. Clin. Med..

[71] Al-Mahmood S., Sapiezynski J., Garbuzenko O.B., Minko T. (2018). Metastatic and Triple-Negative Breast Cancer: Challenges and Treatment Options. Drug Deliv. Transl. Res..

[72] Subramaniyan B., Sridharan S., Howard C.M., Tilley A.M.C., Basuroy T., De La Serna I., Butt E., Raman D. (2020). Role of the CXCR4-LASP1 Axis in the Stabilization of Snail1 in Triple-Negative Breast Cancer. Cancers.

[73] Liaghat M., Ferdousmakan S., Mortazavi S.H., Yahyazadeh S., Irani A., Banihashemi S., Seyedi Asl F.S., Akbari A., Farzam F., Aziziyan F. (2024). The Impact of Epithelial-Mesenchymal Transition (EMT) Induced by Metabolic Processes and Intracellular Signaling Pathways on Chemo-Resistance, Metastasis, and Recurrence in Solid Tumors. Cell Commun. Signal..

[74] Grasset E.M., Dunworth M., Sharma G., Loth M., Tandurella J., Cimino-Mathews A., Gentz M., Bracht S., Haynes M., Fertig E.J. (2022). Triple-Negative Breast Cancer Metastasis Involves Complex Epithelial-Mesenchymal Transition Dynamics and Requires Vimentin. Sci. Transl. Med..

[75] Cojoc M., Peitzsch C., Trautmann F., Polishchuk L., Telegeev G.D., Dubrovska A. (2013). Emerging Targets in Cancer Management: Role of the CXCL12/CXCR4 Axis. OncoTargets Ther..

[76] Huang P., Zhang X., Prabhu J.S., Pandey V. (2024). Therapeutic Vulnerabilities in Triple Negative Breast Cancer: Stem-like Traits Explored within Molecular Classification. Biomed. Pharmacother..

[77] Ma Z., Zhou F., Jin H., Wu X. (2024). Crosstalk between CXCL12/CXCR4/ACKR3 and the STAT3 Pathway. Cells.

[78] Song K., Farzaneh M. (2021). Signaling Pathways Governing Breast Cancer Stem Cells Behavior. Stem Cell Res. Ther..

[79] Mengistu B.A., Tsegaw T., Demessie Y., Getnet K., Bitew A.B., Kinde M.Z., Beirhun A.M., Mebratu A.S., Mekasha Y.T., Feleke M.G. (2024). Comprehensive Review of Drug Resistance in Mammalian Cancer Stem Cells: Implications for Cancer Therapy. Cancer Cell Int..

[80] Sun H.-R., Wang S., Yan S.-C., Zhang Y., Nelson P.J., Jia H.-L., Qin L.-X., Dong Q.-Z. (2019). Therapeutic Strategies Targeting Cancer Stem Cells and Their Microenvironment. Front. Oncol..

[81] Domanska U.M., Kruizinga R.C., Nagengast W.B., Timmer-Bosscha H., Huls G., De Vries E.G.E., Walenkamp A.M.E. (2013). A Review on CXCR4/CXCL12 Axis in Oncology: No Place to Hide. Eur. J. Cancer.

[82] Wei R., Liu S., Zhang S., Min L., Zhu S. (2020). Cellular and Extracellular Components in Tumor Microenvironment and Their Application in Early Diagnosis of Cancers. Anal. Cell. Pathol..

[83] Ma Z., Yu D., Tan S., Li H., Zhou F., Qiu L., Xie X., Wu X. (2025). CXCL12 Alone Is Enough to Reprogram Normal Fibroblasts into Cancer-Associated Fibroblasts. Cell Death Discov..

[84] Chen Y., Ramjiawan R.R., Reiberger T., Ng M.R., Hato T., Huang Y., Ochiai H., Kitahara S., Unan E.C., Reddy T.P. (2015). CXCR4 Inhibition in Tumor Microenvironment Facilitates Anti-programmed Death Receptor-1 Immunotherapy in Sorafenib-treated Hepatocellular Carcinoma in Mice. Hepatology.

[85] Zeng Y., Li B., Liang Y., Reeves P.M., Qu X., Ran C., Liu Q., Callahan M.V., Sluder A.E., Gelfand J.A. (2019). Dual Blockade of CXCL12-CXCR4 and PD-1–PD-L1 Pathways Prolongs Survival of Ovarian Tumor–Bearing Mice by Prevention of Immunosuppression in the Tumor Microenvironment. FASEB J..

[86] Chaudary N., Hill R.P., Milosevic M. (2024). Targeting the CXCL12/CXCR4 Pathway to Reduce Radiation Treatment Side Effects. Radiother. Oncol..

[87] Liu Y.-T., Sun Z.-J. (2021). Turning Cold Tumors into Hot Tumors by Improving T-Cell Infiltration. Theranostics.

[88] Chen Z., Han F., Du Y., Shi H., Zhou W. (2023). Hypoxic Microenvironment in Cancer: Molecular Mechanisms and Therapeutic Interventions. Signal Transduct. Target. Ther..

[89] Martin S.K., Diamond P., Williams S.A., To L.B., Peet D.J., Fujii N., Gronthos S., Harris A.L., Zannettino A.C.W. (2010). Hypoxia-Inducible Factor-2 Is a Novel Regulator of Aberrant CXCL12 Expression in Multiple Myeloma Plasma Cells. Haematologica.

[90] Shibue T., Weinberg R.A. (2017). EMT, CSCs, and Drug Resistance: The Mechanistic Link and Clinical Implications. Nat. Rev. Clin. Oncol..

[91] Liu Y., Liang J., Zhang Y., Guo Q. (2024). Drug Resistance and Tumor Immune Microenvironment: An Overview of Current Understandings (Review). Int. J. Oncol..

[92] Conley-LaComb M.K., Semaan L., Singareddy R., Li Y., Heath E.I., Kim S., Cher M.L., Chinni S.R. (2016). Pharmacological Targeting of CXCL12/CXCR4 Signaling in Prostate Cancer Bone Metastasis. Mol. Cancer.

[93] Misra A.C., Luker K.E., Durmaz H., Luker G.D., Lahann J. (2015). CXCR4-Targeted Nanocarriers for Triple Negative Breast Cancers. Biomacromolecules.

[94] Chittasupho C., Anuchapreeda S., Sarisuta N. (2017). CXCR4 Targeted Dendrimer for Anti-Cancer Drug Delivery and Breast Cancer Cell Migration Inhibition. Eur. J. Pharm. Biopharm..

[95] Sun X., Cheng G., Hao M., Zheng J., Zhou X., Zhang J., Taichman R.S., Pienta K.J., Wang J. (2010). CXCL12/CXCR4/CXCR7 Chemokine Axis and Cancer Progression. Cancer Metastasis Rev..

[96] Giancotti F.G. (2013). Mechanisms Governing Metastatic Dormancy and Reactivation. Cell.

[97] Wang K., Wang C., Yang H., Chen G., Wang K., Ji P., Sun X., Fan X., Ma J., Cui Z. (2025). A Dual-Targeting Peptide–Drug Conjugate Based on CXCR4 and FOLR1 Inhibits Triple-Negative Breast Cancer. Acta Pharm. Sin. B.

[98] Daniel S.K., Seo Y.D., Pillarisetty V.G. (2020). The CXCL12-CXCR4/CXCR7 Axis as a Mechanism of Immune Resistance in Gastrointestinal Malignancies. Semin. Cancer Biol..

[99] Karpova D., Dauber K., Spohn G., Chudziak D., Wiercinska E., Schulz M., Pettit A.R., Levesque J.P., Romagnoli B., Patel K. (2013). The Novel CXCR4 Antagonist POL5551 Mobilizes Hematopoietic Stem and Progenitor Cells with Greater Efficiency than Plerixafor. Leukemia.

[100] Lu G., Qiu Y., Su X. (2021). Targeting CXCL12-CXCR4 Signaling Enhances Immune Checkpoint Blockade Therapy Against Triple Negative Breast Cancer. Eur. J. Pharm. Sci..

[101] Liu Y., Chen X., Zhang W., Yu B., Cen Y., Liu Q., Tang Y., Li S. (2025). A CXCR4-Targeted Immunomodulatory Nanomedicine for Photodynamic Amplified Immune Checkpoint Blockade Therapy against Breast Cancer. Acta Biomater..

[102] Pernas S., Martin M., Kaufman P.A., Gil-Martin M., Gomez Pardo P., Lopez-Tarruella S., Manso L., Ciruelos E., Perez-Fidalgo J.A., Hernando C. (2018). Balixafortide plus Eribulin in HER2-Negative Metastatic Breast Cancer: A Phase 1, Single-Arm, Dose-Escalation Trial. Lancet Oncol..

[103] Steurer M., Montillo M., Scarfò L., Mauro F.R., Andel J., Wildner S., Trentin L., Janssens A., Burgstaller S., Frömming A. (2019). Olaptesed Pegol (NOX-A12) with Bendamustine and Rituximab: A Phase IIa Study in Patients with Relapsed/Refractory Chronic Lymphocytic Leukemia. Haematologica.

[104] Wu Y., Zhou T., Qian D., Liu X., Xu Y., Hong W., Meng X., Tang H. (2023). Z-Guggulsterone Induces Cell Cycle Arrest and Apoptosis by Targeting the P53/CCNB1/PLK1 Pathway in Triple-Negative Breast Cancer. ACS Omega.

[105] Gupta A., Nishchaya K., Saha M., Naik G.A.R.R., Yadav S., Srivastava S., Roy A.A., Moorkoth S., Mutalik S., Dhas N. (2024). Recent Advancements in Nanoconstructs for the Theranostics Applications for Triple Negative Breast Cancer. J. Drug Deliv. Sci. Technol..

[106] Wu B., Zhang B., Li B., Wu H., Jiang M. (2024). Cold and Hot Tumors: From Molecular Mechanisms to Targeted Therapy. Signal Transduct. Target. Ther..

[107] Long L., Fei X., Chen L., Yao L., Lei X. (2024). Potential Therapeutic Targets of the JAK2/STAT3 Signaling Pathway in Triple-Negative Breast Cancer. Front. Oncol..

[108] Massihnia D., Galvano A., Fanale D., Perez A., Castiglia M., Incorvaia L., Listì A., Rizzo S., Cicero G., Bazan V. (2016). Triple Negative Breast Cancer: Shedding Light onto the Role of Pi3k/Akt/Mtor Pathway. Oncotarget.

[109] Pusic I., DiPersio J.F. (2010). Update on Clinical Experience with AMD3100, an SDF-1/CXCL12–CXCR4 Inhibitor, in Mobilization of Hematopoietic Stem and Progenitor Cells. Curr. Opin. Hematol..

[110] Russell N., Douglas K., Ho A.D., Mohty M., Carlson K., Ossenkoppele G.J., Milone G., Pareja M.O., Shaheen D., Willemsen A. (2013). Plerixafor and Granulocyte Colony-Stimulating Factor for First-Line Steady-State Autologous Peripheral Blood Stem Cell Mobilization in Lymphoma and Multiple Myeloma: Results of the Prospective PREDICT Trial. Haematologica.

[111] Ciavattone N.G., Bevoor A., Farfel A., Rehman A., Ho K.K.Y., Rock E.C., Chen Y.-C., Luker K.E., Humphries B.A., Luker G.D. (2025). Inhibiting CXCR4 Reduces Immunosuppressive Effects of Myeloid Cells in Breast Cancer Immunotherapy. Sci. Rep..

[112] Gil-Martin M., Gomez Pardo P., Lopez-Tarruella S., Manso L., Perez-Fidalgo J.A., Ademuyiwa F.O., Mayer I.A., Pluard T.J., Martinez Garcia M., Kaufman P.A. (2017). Phase I Study of the Combination of Balixafortide (CXCR4 Inhibitor) and Eribulin in HER2-Negative Metastatic Breast Cancer (MBC) Patients (Pts). J. Clin. Oncol..

[113] Ghobrial I.M., Liu C.-J., Redd R.A., Perez R.P., Baz R., Zavidij O., Sklavenitis-Pistofidis R., Richardson P.G., Anderson K.C., Laubach J. (2020). A Phase Ib/II Trial of the First-in-Class Anti-CXCR4 Antibody Ulocuplumab in Combination with Lenalidomide or Bortezomib Plus Dexamethasone in Relapsed Multiple Myeloma. Clin. Cancer Res..

[114] Liu G., Chen T., Zhang X., Hu B., Shi H. (2024). Immune Checkpoint Inhibitor-Associated Cardiovascular Toxicities: A Review. Heliyon.

[115] Cambier S., Gouwy M., Proost P. (2023). The Chemokines CXCL8 and CXCL12: Molecular and Functional Properties, Role in Disease and Efforts towards Pharmacological Intervention. Cell. Mol. Immunol..

[116] Liu B., Zhou H., Tan L., Siu K.T.H., Guan X.-Y. (2024). Exploring Treatment Options in Cancer: Tumor Treatment Strategies. Signal Transduct. Target. Ther..

[117] Duda D.G., Kozin S.V., Kirkpatrick N.D., Xu L., Fukumura D., Jain R.K. (2011). CXCL12 (SDF1α)-CXCR4/CXCR7 Pathway Inhibition: An Emerging Sensitizer for Anticancer Therapies?. Clin. Cancer Res..

[118] Chen X., Feng L., Huang Y., Wu Y., Xie N. (2022). Mechanisms and Strategies to Overcome PD-1/PD-L1 Blockade Resistance in Triple-Negative Breast Cancer. Cancers.

[119] Said S.S., Ibrahim W.N. (2024). Breaking Barriers: The Promise and Challenges of Immune Checkpoint Inhibitors in Triple-Negative Breast Cancer. Biomedicines.

[120] Imani S., Farghadani R., Roozitalab G., Maghsoudloo M., Emadi M., Moradi A., Abedi B., Jabbarzadeh Kaboli P. (2025). Reprogramming the Breast Tumor Immune Microenvironment: Cold-to-Hot Transition for Enhanced Immunotherapy. J. Exp. Clin. Cancer Res..

[121] Brown J.S., O’Carrigan B., Jackson S.P., Yap T.A. (2017). Targeting DNA Repair in Cancer: Beyond PARP Inhibitors. Cancer Discov..

[122] Khan M.A., Srivastava S.K., Zubair H., Patel G.K., Arora S., Khushman M., Carter J.E., Gorman G.S., Singh S., Singh A.P. (2020). Co-Targeting of CXCR4 and Hedgehog Pathways Disrupts Tumor-Stromal Crosstalk and Improves Chemotherapeutic Efficacy in Pancreatic Cancer. J. Biol. Chem..

[123] Gagner J.-P., Sarfraz Y., Ortenzi V., Alotaibi F.M., Chiriboga L.A., Tayyib A.T., Douglas G.J., Chevalier E., Romagnoli B., Tuffin G. (2017). Multifaceted C-X-C Chemokine Receptor 4 (CXCR4) Inhibition Interferes with Anti–Vascular Endothelial Growth Factor Therapy–Induced Glioma Dissemination. Am. J. Pathol..

[124] Zhao R., Liu J., Li Z., Zhang W., Wang F., Zhang B. (2022). Recent Advances in CXCL12/CXCR4 Antagonists and Nano-Based Drug Delivery Systems for Cancer Therapy. Pharmaceutics.

[125] Meng Y., Zhou J., Liu X., Zeng F., Wen T., Meng J., Liu J., Xu H. (2023). CXC Chemokine Receptor Type 4 Antagonistic Gold Nanorods Induce Specific Immune Responses and Long-Term Immune Memory to Combat Triple-Negative Breast Cancer. ACS Appl. Mater. Interfaces.

[126] Marciscano A.E., Anandasabapathy N. (2021). The Role of Dendritic Cells in Cancer and Anti-Tumor Immunity. Semin. Immunol..

[127] Huang Y., Wang K., Yu M., Zhou Q., Wang J., Chen S., Gong J., Yang M., Huang J., Zhao Y. (2025). Co-Delivery Paclitaxel and IR783 as Nanoparticles for Potentiated Chemo-Photothermal-Immunotherapy of Triple-Negative Breast Cancer. Mater. Today Bio.

[128] Wang S., Liu J., Cui Y., Sun M., Wang W., Chen J., Gu J., Yang Z. (2025). Macrophage-Centered Therapy Strategies: A Promising Weapon in Cancer Immunotherapy. Asian J. Pharm. Sci..

[129] Cortés J., Holgado E., Perez-Garcia J. (2018). CXCR4 Antagonists for Treatment of Breast Cancer. Oncotarget.

[130] Barbari C., Fontaine T., Parajuli P., Lamichhane N., Jakubski S., Lamichhane P., Deshmukh R.R. (2020). Immunotherapies and Combination Strategies for Immuno-Oncology. Int. J. Mol. Sci..

[131] He M., Hao S., Ma L., Xiu B., Yang B., Wang Z., Xue J., Chi Y., Xiong M., Chen J. (2024). Neoadjuvant Anthracycline Followed by Toripalimab Combined with Nab-Paclitaxel in Patients with Early Triple-Negative Breast Cancer (NeoTENNIS): A Single-Arm, Phase II Study. eClinicalMedicine.

[132] Li Z., Wang Y., Shen Y., Qian C., Oupicky D., Sun M. (2020). Targeting Pulmonary Tumor Microenvironment with CXCR4-Inhibiting Nanocomplex to Enhance Anti–PD-L1 Immunotherapy. Sci. Adv..

[133] Jain A., Stebbing J. (2025). The Relationship Between Response Rate and Survival Benefits in Randomized Immunotherapy Studies. Cancers.

[134] Ribeiro R., Carvalho M.J., Goncalves J., Moreira J.N. (2022). Immunotherapy in Triple-Negative Breast Cancer: Insights into Tumor Immune Landscape and Therapeutic Opportunities. Front. Mol. Biosci..

[135] Xiang J., Hurchla M.A., Fontana F., Su X., Amend S.R., Esser A.K., Douglas G.J., Mudalagiriyappa C., Luker K.E., Pluard T. (2015). CXCR4 Protein Epitope Mimetic Antagonist POL5551 Disrupts Metastasis and Enhances Chemotherapy Effect in Triple-Negative Breast Cancer. Mol. Cancer Ther..

[136] Evans T.R.J., Dean E., Molife L.R., Lopez J., Ranson M., El-Khouly F., Zubairi I., Savulsky C., Reyderman L., Jia Y. (2019). Phase 1 Dose-Finding and Pharmacokinetic Study of Eribulin-Liposomal Formulation in Patients with Solid Tumours. Br. J. Cancer.

[137] Korn R.L., Crowley J.J. (2013). Overview: Progression-Free Survival as an Endpoint in Clinical Trials with Solid Tumors. Clin. Cancer Res..

[138] Zhu S., Wu Y., Song B., Yi M., Yan Y., Mei Q., Wu K. (2023). Recent Advances in Targeted Strategies for Triple-Negative Breast Cancer. J. Hematol. Oncol..

[139] Malik J.A., Ahmed S., Jan B., Bender O., Al Hagbani T., Alqarni A., Anwar S. (2022). Drugs Repurposed: An Advanced Step towards the Treatment of Breast Cancer and Associated Challenges. Biomed. Pharmacother..

[140] Zhang F., Ma Y., Li D., Wei J., Chen K., Zhang E., Liu G., Chu X., Liu X., Liu W. (2024). Cancer Associated Fibroblasts and Metabolic Reprogramming: Unraveling the Intricate Crosstalk in Tumor Evolution. J. Hematol. Oncol..

[141] Spiotto M., Fu Y.-X., Weichselbaum R.R. (2016). The Intersection of Radiotherapy and Immunotherapy: Mechanisms and Clinical Implications. Sci. Immunol..

[142] Ruiz-Martínez S., Ribas X., Costas M., Landberg G., Puig T. (2025). Characterization and Targeting of Chemoresistant Triple-Negative Breast Cancer Subtypes Using Amino-Pyridine Compounds. Biochim. Et Biophys. Acta (BBA)-Mol. Basis Dis..

[143] Takaki H., Cornelis F.H. (2018). Can the Combination of Ablation and Immunomodulation Become the Breakthrough of Cancer Treatment?. Diagn. Interv. Imaging.

[144] Ziyao L., Jingzhe W., Huabiao C. (2021). CXCR4 Antagonist AMD3100 (Plerixafor) Modulates Immune Responses in the Tumor Microenvironment. Int. J. Cancer Clin. Res..

[145] Sahai E., Astsaturov I., Cukierman E., DeNardo D.G., Egeblad M., Evans R.M., Fearon D., Greten F.R., Hingorani S.R., Hunter T. (2020). A Framework for Advancing Our Understanding of Cancer-Associated Fibroblasts. Nat. Rev. Cancer.

[146] Choueiri T.K., Atkins M.B., Rose T.L., Alter R.S., Ju Y., Niland K., Wang Y., Arbeit R., Parasuraman S., Gan L. (2021). A Phase 1b Trial of the CXCR4 Inhibitor Mavorixafor and Nivolumab in Advanced Renal Cell Carcinoma Patients with No Prior Response to Nivolumab Monotherapy. Investig. New Drugs.

[147] Bockorny B., Semenisty V., Macarulla T., Borazanci E., Wolpin B.M., Stemmer S.M., Golan T., Geva R., Borad M.J., Pedersen K.S. (2020). BL-8040, a CXCR4 Antagonist, in Combination with Pembrolizumab and Chemotherapy for Pancreatic Cancer: The COMBAT Trial. Nat. Med..

[148] Huang E.H., Singh B., Cristofanilli M., Gelovani J., Wei C., Vincent L., Cook K.R., Lucci A. (2009). A CXCR4 Antagonist CTCE-9908 Inhibits Primary Tumor Growth and Metastasis of Breast Cancer. J. Surg. Res..

[149] Masrour M., Moeinafshar A., Poopak A., Razi S., Rezaei N. (2025). The Role of CXC Chemokines and Receptors in Breast Cancer. Clin. Exp. Med..

[150] Chuan T., Li T., Yi C. (2020). Identification of CXCR4 and CXCL10 as Potential Predictive Biomarkers in Triple Negative Breast Cancer (TNBC). Med. Sci. Monit..

[151] Liu S., Xie S.M., Liu W., Gagea M., Hanker A.B., Nguyen N., Raghavendra A.S., Yang-Kolodji G., Chu F., Neelapu S.S. (2023). Targeting CXCR4 Abrogates Resistance to Trastuzumab by Blocking Cell Cycle Progression and Synergizes with Docetaxel in Breast Cancer Treatment. Breast Cancer Res..

[152] Lüönd F., Tiede S., Christofori G. (2021). Breast Cancer as an Example of Tumour Heterogeneity and Tumour Cell Plasticity during Malignant Progression. Br. J. Cancer.

[153] Hamshaw I., Cominetti M.M.D., Lai W.-Y., Searcey M., Mueller A. (2023). The Development of Potent, Competitive CXCR4 Antagonists for the Prevention of Cancer Metastasis. Biochem. Pharmacol..

[154] Ma L., Guo H., Zhao Y., Liu Z., Wang C., Bu J., Sun T., Wei J. (2024). Liquid Biopsy in Cancer: Current Status, Challenges and Future Prospects. Signal Transduct. Target. Ther..

[155] Yang J., Tian E., Chen L., Liu Z., Ren Y., Mao W., Zhang Y., Zhang J. (2024). Development and Therapeutic Perspectives of CXCR4 Antagonists for Disease Therapy. Eur. J. Med. Chem..

[156] Zhou M., Luo C., Zhou Z., Li L., Huang Y. (2021). Improving Anti-PD-L1 Therapy in Triple Negative Breast Cancer by Polymer-Enhanced Immunogenic Cell Death and CXCR4 Blockade. J. Control. Release.

[157] Zhang M.-R., Fang L.-L., Guo Y., Wang Q., Li Y.-J., Sun H.-F., Xie S.-Y., Liang Y. (2024). Advancements in Stimulus-Responsive Co-Delivery Nanocarriers for Enhanced Cancer Immunotherapy. Int. J. Nanomed..

[158] Cho B.-S., Kim H.-J., Konopleva M. (2017). Targeting the CXCL12/CXCR4 Axis in Acute Myeloid Leukemia: From Bench to Bedside. Korean J. Intern. Med..

[159] Singh D.D., Haque S., Kim Y., Han I., Yadav D.K. (2024). Remodeling of tumour microenvironment: Strategies to overcome therapeutic resistance and innovate immunoengineering in triple-negative breast cancer. Front. Immunol..

[160] Singh D.D., Lee H.J., Yadav D.K. (2023). Recent Clinical Advances on Long Non-Coding RNAs in Triple-Negative Breast Cancer. Cells.

[161] Singh D.D., Lee H.J., Yadav D.K. (2022). Clinical updates on tyrosine kinase inhibitors in HER2-positive breast cancer. Front. Pharmacol..

[162] Singh D.D., Han I., Choi E.H., Yadav D.K. (2021). CRISPR/Cas9 based genome editing for targeted transcriptional control in triple-negative breast cancer. Comput. Struct. Biotechnol. J..

